# An oxygen gradient chamber with simple geometry for generating oxygen gradients in hydrogel-based cell cultures within a well-plate-compatible format

**DOI:** 10.3389/fbioe.2026.1937577

**Published:** 2026-10-01

**Authors:** Tabea Marie Fleischhammer, Nele Bösing, Janina Bahnemann, Iliyana Pepelanova, Antonina Lavrentieva

**Affiliations:** 1 Institute of Technical Chemistry, Leibniz University Hannover, Hanover, Germany; 2 Institute of Physics, University of Augsburg, Augsburg, Germany; 3 Centre for Advanced Analytics and Predictive Sciences (CAAPS), University of Augsburg, Augsburg, Germany

**Keywords:** 3D cell culture, 3D oxygen gradients, GelMA, hydrogels, hypoxia, hypoxia-activated prodrug, oxygen gradients, tirapazamine

## Abstract

Oxygen gradients strongly influence cellular behaviour in 3D tissues, but their controlled implementation in standard cell culture remains technically challenging. Here, we present a simple 3D-printed oxygen gradient chamber for hydrogel-based cell culture. The system uses a screw-mounted glass cover to locally restrict oxygen exchange between the gelatine methacryloyl (GelMA) hydrogel and its surrounding medium, thereby generating a diffusion- and consumption-driven radial oxygen gradient without pumps, gas channels, introduction of additional chemicals or complex microfluidics. Hypoxia reporter mesenchymal stem cells embedded within the GelMA hydrogel showed distinct hypoxia sensor signalling underneath the glass-covered region after 24 h. Direct oxygen measurements confirmed gradient formation, with oxygen levels stabilising around 2% O_2_ in the middle of the construct and increasing to 5%–6% O_2_ in its peripheral regions. As a proof of concept, the hypoxia-activated prodrug tirapazamine was applied in 2D cell culture, 3D hydrogel discs of two different stiffnesses and within the oxygen gradient chamber. The results showed oxygen-dependent toxicity in 2D MSC culture, with IC_50_ values of 148.7 μM at atmospheric oxygen and 20.4 μM at 2.5% oxygen. In 3D hydrogel discs, the calculated IC_50_ value at atmospheric oxygen was >400 µM and 47.3 μM at 2.5% oxygen. In the oxygen gradient chamber, tirapazamine-induced cell death was enhanced in the central hypoxic region. Thus, the presented chamber provides an accessible and well-plate-compatible platform of simple geometry for studying spatiotemporal effects of hypoxia and oxygen-dependent drug effects in 3D hydrogel cultures.

## 1 Introduction

Oxygen availability is a key regulator of cellular physiology, tissue homeostasis and disease progression. *In vivo*, cells are rarely exposed to atmospheric oxygen levels and local oxygen tensions vary depending on tissue vascularisation levels, the diffusion distances between the cells and their surrounding vessels, as well as the cell-type specific oxygen consumption rate (Carreau et al., 2011; Ivanovic, 2009; Malda et al., 2007). This spatial oxygen heterogeneity is relevant in both physiological and pathological contexts, including embryonic development, stem cell regulation, wound healing, inflammation or progression of solid tumours (Al Tameemi et al., 2019; Carreau et al., 2011; Höckel and Vaupel, 2001; Keith and Simon, 2007; Malda et al., 2007; Simon and Keith, 2008). Therefore, *in vitro* systems that reproduce defined oxygen gradients are valuable tools for studying oxygen-dependent cell behaviour and drug responses under more physiologically relevant conditions.

Three-dimensional (3D) hydrogel cultures are well-suited for modelling the *in vivo* cellular microenvironment as they provide cell-matrix interactions, mechanical cues and diffusion-limited transport conditions absent in conventional two-dimensional (2D) monolayer culture (Kapałczyńska et al., 2018; Pampaloni et al., 2007). Hydrogels are especially useful for 3D cell culture because their water-rich polymer networks can mimic some features of the extracellular matrix while allowing adjustment of material stiffness, composition and degradation behaviour (Catoira et al., 2019; Malda et al., 2013; Peppas and Hoffman, 2013; Schuurman et al., 2013). However, O_2_ availability in 3D constructs is governed by the equilibrium between diffusion transfer and cellular consumption; therefore, it is affected by the construct’s geometry, seeded cell density and overall hydrogel properties (Malda et al., 2007; Schmitz et al., 2020; 2022; Wolff et al., 2019). Combining 3D culture with controlled oxygen gradients is therefore of particular interest for modelling of tumour hypoxia under physioxic or hypoxic conditions and when investigating oxygen-dependent drug responses. Nevertheless, the implementation of reproducible, accessible and easy-to-use oxygen gradients in 3D cell culture remains technically challenging (Palacio-Castañeda et al., 2022).

Several strategies have been developed to generate oxygen gradients *in vitro*. Gas-driven microfluidic systems can generate defined oxygen profiles with gas channels or gas-permeable membranes. They have been successfully used to study oxygen-dependent signalling, migration and angiogenesis in 2D and 3D culture systems (Allen and Bhatia, 2003; Funamoto et al., 2012; Hsu et al., 2023; Koens et al., 2020; Orabi et al., 2025; Rexius-Hall et al., 2014; Sato et al., 2014). Other approaches generate gradients by spatially confined chemical oxygen scavenging by introduced chemicals or the hydrogel itself or by combining cellular oxygen consumption with diffusion barriers (Ando et al., 2017; Blatchley et al., 2021; Chang et al., 2014; Chen et al., 2011; Park and Gerecht, 2014; Volkmer et al., 2008). Cell-density gradients and rolled or thick hydrogel constructs have also been used to generate oxygen gradients through cell-mediated oxygen consumption and extended diffusion distances (Cheema et al., 2008; Volkmer et al., 2008). These systems have provided important biological insights and demonstrated that oxygen gradients can influence cell signalling, migration, viability and drug response. However, many existing platforms require specialised fabrication procedures, gas or pump connections, complex microfluidic architectures, introduction of additional chemical components or highly material-specific chemistries (Boyce et al., 2019; Palacio-Castañeda et al., 2020; Park and Gerecht, 2014). Our group previously also reported the development of an oxygen gradient system for 3D hydrogels; however, its implementation required active air perfusion and the use of a hypoxia incubator (Schmitz et al., 2022). These requirements can limit routine use in standard cell culture laboratories, as assays should best be compatible with conventional culture ware and readout formats while still enabling stable culture over a biologically meaningful time.

Here, we present a simple and low-cost oxygen-gradient chamber for hydrogel-based 3D cell culture. The system consists of a 3D-printed bottom frame serving as a hydrogel mould and a screw-mounted top part that fixes a glass slide onto the central region of the hydrogel. The glass locally restricts oxygen exchange between the hydrogel and the surrounding medium, while the uncovered peripheral regions remain exposed to oxygen supplied from the culture medium. In combination with the cellular oxygen consumption, this geometry generates a bidirectional oxygen gradient without requiring pumps, gas channels, oxygen scavengers or complex microfluidics. We characterised the chamber using GelMA hydrogels containing mesenchymal stem cells (MSCs). Gradient formation was assessed by direct oxygen measurements using oxygen-sensitive sensor foils and by hypoxia-reporter MSCs expressing the fluorescent protein UnaG upon HIF-1 stabilisation. As a functional proof of concept, we evaluated tirapazamine toxicity in 2D culture, 3D GelMA hydrogel discs and the oxygen-gradient chamber. Finally, we suggest design variants that allow the covered area to be modified and provide a basis for future implementation of orthogonal gradients. Together, this study introduces a low-complexity and well-plate-compatible platform for generating and analysing oxygen gradients in hydrogel-based 3D cell culture.

## 2 Materials and methods

### 2.1 Chamber design, manufacturing and post-processing

A computer-aided design (CAD) model was created using Solidworks 2024 (Dassault Systèmes Deutschland GmbH, Stuttgart, Germany) and saved as an.STL file for printing. All.STL files are provided as Supplementary Material.

The chambers’ top and bottom constructs were fabricated via MultiJet 3D printing with a ProJet® MJP 2500 Plus (3D Systems GmbH; Mörsfelden-Walldorf, Germany) from M2-RCL printing material and M2-SUP support material (3D Systems GmbH, Mörsfelden-Walldorf, Germany). Following printing, the build platform was cooled at −18 °C for 15 min, after which the printed objects were detached from the platform. All pieces were placed in hot steam in EasyClean units (3D Systems GmbH, Mörsfelden-Walldorf, Germany), followed by dissolving residues of the support wax in paraffin oil (Carl Roth GmbH und Co. KG, Karlsruhe, Germany) at 65 °C. Afterwards, the oil was removed by rinsing three times with hot (50 °C) detergent (Procter and Gamble Germany Holding GmbH, Schwalbach am Taunus, Germany) solution in an ultrasound bath (Elmasonic S30; Schmidbauer GmbH, Singen, Germany) for a minimum of 30 min, followed by two separate incubations at 60 °C with deionised water for 20 min.

After the printed constructs had fully dried, two droplets of UV-curable adhesive (Norland Optical Adhesive 72; Norland Products Inc., Cranbury, United States) were thinly spread on the bottom construct and the construct was glued onto the middle of a conventional cell culture 6-well plate (Sarstedt, Nümbrecht, Germany) for adherent cells. The optical adhesive was cured at 365 nm with 1.5 mW/cm^2^ for 30 min using a UV lamp (VL-6.LC UV lamp 365/254 nm 6 W; Vilber Lourmat, Collégien, France). The edge between the outer corner of the bottom construct and the cell culture plate bottom was additionally sealed with a silicone glue (Silicone Rubber compound flowable fluid; RS Components GmbH, Frankfurt am Main, Germany) which was left to dry for 24 h. Glass plates were cut from conventional microscopy slides (1 mm thickness; VWR International, Leuven, Belgium), using a sliding calliper and a sharp cemented carbide tip to scratch the glass surface at the defined width of 6 or 8 mm, followed by breakage of the glass over the edge of an engineer’s square, resulting in a dimension of 1 × 6 × 27 mm per piece.

Disinfection of the chamber parts was performed using 70% v/v isopropanol in an ultrasound bath (SONOREX SUPER RK 100 H; Bandelin, Berlin, Germany) followed by an additional 20 min under the aforementioned UV lamp at 365 nm after aspiration of the isopropanol. Top constructs, glass plates, stainless steel M2 screws and the hex key were sterilised in the same manner but without using an ultrasound bath.

### 2.2 GelMA hydrogel synthesis, characterisation and preparation

Low and high degree of functionalisation (DoF) gelatine methacryloyl hydrogels were synthesised using the one-pot synthesis protocol of Shirahama *et al.* from type A porcine skin gelatine (260 bloom for high and low DoF batch with 29.3%; GELITA AG; Eberbach, Germany; 300 bloom for low DoF batch with 21.5% DoF; Sigma Aldrich Chemie GmbH, Munich, Germany) (Shirahama et al., 2016). 0.04 mL methacrylic anhydride (MAA; Sigma-Aldrich Chemie GmbH; Taufkirchen, Germany) per g of gelatine was used for the synthesis of low DoF GelMA and 0.1 mL MAA per g of gelatine were used for the high DoF batch, respectively. The low DoF materials had a DoF of 29.3% and 21.5%, respectively, while the high DoF material had a DoF of 99.6%, as determined by 2,4,6-trinitrobenzene sulfonic acid (TNBS) assay (Shirahama et al., 2016).

Hydrogels were prepared at a final concentration of 7.5% w/v (low DoF) or 5% w/v (high DoF) by solubilising the desired lyophilised hydrogel amount at 37 °C in PBS until complete dissolution. The solution was sterile filtered through a syringe filter with a 0.45 µm pore size and polyether sulfone (PES) membrane (qpore® via NeoLab GmbH, Heidelberg, Germany). Lastly, lithium phenyl-2,4,6-trimethylbenzoylphosphinate (LAP; Tokyo Chemical Industry, Zwijndrecht, Belgium) dissolved in phosphate-buffered saline (PBS) was added as the photoinitiator at a final concentration of 0.1% w/v.

### 2.3 Oscillatory rheology

Oscillatory rheology with a MCR 302 Modular Rheometer (Anton Paar GmbH, Baden-Württemberg, Germany), equipped with a parallel-plate geometry with a diameter of 20 mm was used to determine material stiffness. Storage and loss moduli were evaluated using a time sweep oscillatory test with a strain amplitude of 1% and a frequency of 1 Hz with the plate temperature set to 37 °C. The gap width was 0.6 mm, leading to a sample volume of 200 µL. Samples were exposed to ultraviolet (UV) light at 365 nm during the first 5 min of each measurement, followed by an additional 5 min without UV irradiation.

### 2.4 Isolation and routine culture of primary mesenchymal stem cells

Primary human adipose-derived mesenchymal stem cells (hAD-MSC, in the following abbreviated as MSC) were isolated from adipose tissue after abdominoplasty applying the protocol of Zhu *et al.* as previously described (Schmitz et al., 2020; Zhu et al., 2008). Cells from one donor were used in this study. The donor provided their informed consent, as approved by the Institutional Review Board (Hannover Medical School) with the reference number 3475–2017. The obtained cells were characterised by MSC-typical surface markers and functional properties and the presence of adipogenic, chondrogenic, and osteogenic differentiation potential (Dominici et al., 2006; Schmitz et al., 2020).

α-minimum essential medium (α-MEM; Thermo Fisher Scientific, Waltham, MA, United States) was used as the cultivation medium, containing 1 g/L glucose, 2 mM L-glutamine, 10% v/v human serum (CC-pro, Overdorla, Germany) and 50 μg/mL Gentamicin (Merck KGaA, Darmstadt, Germany) and cells were routinely cultivated in a humidified incubator at 37 °C with 5% CO_2_. Subconfluent cells were subcultured twice per week. Briefly, the medium was aspirated, cells were rinsed with PBS and detached with Accutase® for up to 8 min until cells detached from culture ware, followed by pelleting of cells by centrifugation. All centrifugation steps were carried out at 300 × g for 5 min if not noted otherwise. The cell pellet was resuspended in fresh cell culture medium and reseeded at an approximate density of 3,000 cells/cm^2^ after quantification of cell density in a Neubauer type haemocytometer. Cells were routinely tested negative for *mycoplasma*. MSCs and hypoxia reporter MSCs at the passages of 6–13 were used for all experiments.

For the cryopreservation of cells, the culture medium was supplemented with 20% v/v human serum instead of 10% and with an additional 10% v/v of Dimethyl sulfoxide (DMSO; Sigma Aldrich, Munich, Germany).

### 2.5 Generation of hypoxia reporter MSCs

hAD-MSCs were used for lentiviral transduction, to stably integrate the HRE-dUnaG sequence (Erapaneedi et al., 2016) as described in Erapaneedi et al. A detailed protocol of this method can be found in our method book chapter (Fleischhammer et al., 2024).

As an alternative to the cloning process described below, the Addgene plasmid #124372 can be directly purchased. As the lentiviral transfer vector, the *pL-CMV-purom* backbone was used and the cytomegalovirus (CMV) promoter and enhancer were replaced with the amplified *HRE-dUnaG* sequence as described in previous work (Schmitz et al., 2020). Polymerase chain reaction amplification of *HRE-dUnaG* was executed using specific flanking primers with restriction sites for ClaI and BamHI. The *HRE-dUnaG* sequence consists of a minimal human CMV promoter (mCMV), which is preceded by five HRE sequence motifs to drive the expression of the downstream UnaG protein. The C-terminus of UnaG is in frame with a PEST motif as a signal for rapid protein degradation.

The *pL-CMV-purom*-*HRE-dUnaG* vector was used in a second generation lentiviral system in combination with the envelope vector *pMDG* (Addgene plasmid #187440) and the packaging vector p8.91 (Addgene plasmid #187441) to produce lentiviral particles with HEK293T cells (Kindly provided by the Constanca Figueiredo group), using MEXi TM (IBA Lifesciences GmbH, Göttingen, Germany) transfection medium and 20 µL Polyethyleneimine (1 mg/mL; linear, MW 25 000; PEI 25K; Polysciences Europe GmbH, Hirschberg an der Bergstrasse, Germany) as the transfection agent. Viral supernatant was harvested after 48 h, 72 h and 96 h of incubation, sterile filtered with a 0.45 µm PES syringe filter (qpore® via NeoLab GmbH, Heidelberg, Germany). The presence of lentiviral particles was confirmed with a lateral flow assay (Lenti-X GoStix or Lenti-X GoStix Plus; Takara Bio Inc., Kusatsu, Japan).

MSCs were transduced at passage 2 with the viral supernatant. Medium was exchanged with cultivation medium after 6 h of incubation with lentiviral particles and cells were maintained for at least 2 weeks in S2 facilities to ensure that residual lentiviral particles were absent. The supernatant was also tested negative in the aforementioned lateral flow assay for lentiviral particle detection.

Efficient transduction with HRE-dUnaG, presence of stem cell markers, differentiation potential as well as the oxygen-dependent UnaG expression was shown in our previous work (Schmitz et al., 2020). Hypoxia reporter cells were only used for the visualisation of the oxygen gradient. In other experiments, non-transduced MSCs were used.

### 2.6 Cell encapsulation in hydrogels

Encapsulation and cultivation of cells in hydrogels was performed as previously described (Pepelanova et al., 2018). Briefly, cells were harvested as during routine culture and 1 × 10^6^ cells per mL of hydrogel were resuspended in warm hydrogel.

To encapsulate cells in hydrogel discs, hydrogel moulds were poured from room-temperature crosslinking ELASTOSIL® RT 601 A/B silicone (Wacker Chemie AG, Munich, Germany) in a custom-made Teflon mould and cured. The hydrogel mould was disinfected with 70% isopropanol for sterilisation and placed in a Petri dish (ThermoFisher, Waltham, MA, United States) under a laminar flow hood overnight until full liquid evaporation. Each silicone mould has multiple hydrogel wells with a diameter of 6 mm and a 2 mm height. 50 μL of cell-laden hydrogel was transferred per mould and polymerised at 1.2 J/cm^2^ using a UV irradiation system at 365 nm (Bio-Link®; Vilber Lourmat, Collégien, France).

To encapsulate cells in hydrogel in an oxygen gradient chamber, 225 µL of hydrogel was polymerised in the gel mould inside the bottom chamber part at 1.4 J/cm^2^ in the same aforementioned UV curing system.

### 2.7 Assembly of gradient chamber

After the polymerisation of the hydrogels in the bottom part of the chamber, the (optional) 6 mm glass plate and top part were added and fixed with four M2 screws using a sterilised hex key. Afterwards, 7 mL of fresh medium were added, ensuring full submersion of the oxygen gradient chamber within the medium.

### 2.8 Non-invasive oxygen measurements with oxygen sensor foil

A 10 × 10 mm piece of the self-adhesive SF-RPSu4 oxygen measurement foil by PreSens Precision Sensing GmbH (Regensburg, Germany) was glued to the bottom of the gradient chamber directly onto the surface of the 6-well plate.

The VisiSens ScientifiCal 1.0.2.9 software (PreSens Precision Sensing GmbH, Regensburg, Germany) was used and a two-point calibration was carried out at atmospheric oxygen by very thinly covering the foil with cell culture medium and at 0% oxygen in a saturated Na_2_SO_3_ solution in cell culture medium after stabilisation of the resulting measurement. One oxygen gradient chamber with a 6 mm glass slide and the uncovered control sample were monitored every 24 h for a total period of 8 days after assembly. After application of the calibration values, the region of interest (ROI) with oxygen sensor foil was manually cropped from each file and ROI were placed in front of a black background for enhanced contrast.

To yield enough pixels for the data analysis, chamber images were divided in a total of 10 horizontal sections of 1 mm width (see Supplementary Figure S6; sections 3–8 are positioned under the 6 mm wide glass slide) and analysed within the VisiSens software. The mean oxygen concentration of each segment and standard deviation were calculated within the software. As the oxygen sensor foil is not translucent, either the hypoxia reporter cell signal could be microscopically analysed, or the oxygen sensor foil was used to measure the oxygen concentration within the chamber.

### 2.9 Fluorescence microscopy

Imaging of 2D cell culture and 3D hydrogel discs was carried out using the Incucyte S3 (Sartorius AG, Göttingen, Germany). Image acquisition parameters were set as follows: Brightfield: automatic; green channel: 300 ms exposure time, red channel: 400 ms exposure time. For 2D image acquisition, the time-lapse scan function was used; for the acquisition of 3D hydrogel discs, a whole well scan was executed. Instead of using the intrinsic autoscaling function of the Incucyte software during export, the export parameters were adjusted for all experiments. For 2D cell culture experiment: green channel–min: 25, max: 500; red channel: min: 1.4, max: 6.2; For 3D high DoF hydrogel discs with 5% GelMA: green channel–min: 4, max: 1,400; red channel–min: 1.5, max: 5.5; For 3D low DoF hydrogel discs with 7.5% GelMA: green channel–min: 4, max: 1,400; red channel–min: 0.4, max: 3. For 3D high DoF hydrogel discs with 5% GelMA covered with 8 mm glass slide: green channel–min: 16, max: 81; red channel–min: 1.4, max: 6.2.

For the microscopy of the oxygen gradient chamber, a BioTek Cytation 5 Cell Imaging Multimode Reader (Agilent Technologies Inc., Santa Clara, United States) with the following exposure settings within the Gen5 V3.10 software was used: Bright field; light emitting diode (LED) intensity: 4; exposure time: 100 ms; gain: 1.1; GFP channel (469/525 nm): LED-intensity: 5; exposure time: 200 ms; gain: 10. Texas Red channel (586/647 nm): LED-intensity: 10; exposure time: 2,800 ms; gain: 10. Depicted images are either one representative image from a Z-stack with 40 µm distance between Z layers (PI stain) or from a Z-stack projection to a single plane with standard software parameters (hypoxia signalling), which is indicated under the respective graph.

For the display of the hydrogel discs after tirapazamine treatment and for the visualisation of the gradient chambers, the area of the hydrogel was manually cropped after exporting of the whole-well image, and each cropped image was placed in front of a black background for enhanced contrast.

### 2.10 Tirapazamine treatment

For 2D experiments, 6,000 MSCs were seeded per well of a 96-well plate (Sarstedt, Nümbrecht, Germany) the day before incubation with tirapazamine (TPZ, Merck, Germany) and 3D hydrogel discs were prepared as described above.

TPZ was diluted from a stock solution in dimethyl sulfoxide (DMSO) in supplemented cell culture medium at a final concentration of 400 μM, 200 μM, 100 μM, 50 μM, 25 μM, 12.5 µM and 6.25 µM. Medium was aspirated from cells or hydrogels 24 h after seeding and exchanged with the respective TPZ-containing or TPZ-free (mock-treated) medium. Throughout this study, samples cultured in a conventional humidified incubator at 37 °C and 5% CO_2_ without active oxygen control are referred to as “normoxic” samples, describing the non-controlled gas-phase culture condition. Hypoxia samples were incubated for 24 h at 2.5% oxygen in an oxygen control incubator (C16; Labotect Labor-Technik-Göttingen GmbH, Göttingen, Germany), while normoxia samples were incubated in a conventional humidified incubator. To keep sample description as brief as possible, samples that were incubated without additional lowering of the oxygen in the gas phase are “coined” as “normoxic”, even though the resulting oxygen tension within a humidified incubator with 5% CO_2_ are not at atmospheric levels (Wenger et al., 2015).

For the treatment of cells in oxygen gradient chambers, chambers were seeded with MSCs as described above and incubated for 48 h. Half of the samples were then covered with a glass slide and all samples were incubated for 24 h to induce an oxygen gradient in the covered samples before TPZ addition. To minimize potential differences in TPZ diffusion between covered and uncovered regions of hydrogels, chambers were disassembled and incubated with 200 µM TPZ in cell culture medium for 1 h. After incubation, chambers were reassembled and incubated with the TPZ-containing medium for another 24 h before further analysis. A schematic representation of the process is shown in Supplementary Figure S5.

### 2.11 CellTiter-Blue® cell viability assay

After 24 h of TPZ treatment, cells from incubation under reduced oxygen tension (2.5% O_2_) were reoxygenated for 3.5 h at atmospheric oxygen tension in a humidified incubator at 37 °C with 5% CO_2_. Afterwards, the CellTiter-Blue® (CTB; Promega Corporation, Madison, WI, United States) solution was 1:10 diluted with prewarmed unsupplemented α-MEM and 100 µL of this solution were added per well. The CTB test was executed according to the manufacturer’s instructions and six technical replicates were prepared per sample, including a cell-free background control. Cells were incubated for 2.25 h and the fluorescence was measured (Excitation: 544 nm, Emission: 590 nm) with a microplate reader (Fluoroskan Ascent, Thermo Fisher Scientific Inc., Waltham, MA, United States). The mean of the cell-free replicates was subtracted from all measurements of each plate, followed by normalisation to the mean of the mock-treated control samples, resulting in a relative viability to control.

### 2.12 Live/dead cell staining

Medium was aspirated and cells or hydrogels were covered with 1.5 μg/mL propidium iodide (PI; Sigma Aldrich Chemie GmbH, Munich, Germany) and/or 7 µM calcein acetoxymethyl ester (Calcein-AM; Merck KGaA, Darmstadt, Germany) in unsupplemented α-MEM. Cells cultivated in 2D were incubated for 10 min, while 3D hydrogel samples were incubated for 30–40 min. Hydrogels were stained outside of the respective cultivation chamber in a separate well of a 24-well plate (Sarstedt, Nümbrecht, Germany) to reduce staining solution volume.

### 2.13 Analysis of 3D microscopy data

For the analysis of hydrogel discs, the number of propidium iodide stained cells per area was calculated from three different areas of 500 x 500 pixel (px) size per hydrogel from a total of three different hydrogel replicates per TPZ concentration. For the automated analysis, Fiji/ImageJ 1.54p was used. First, a “top hat” filter (r = 3 px), then a “variance” filter (r = 1 px) and lastly a “median” filter (r = 3 px) were used. Then, objects were highlighted with “find edges”, followed by a “despeckle” action. Lastly, images were binarised and particles between the size of 10–100 px^2^ and with a circularity of ≥0.3 were counted, including “hollow” particles.

For the analysis of segments within the oxygen gradient chamber, each chamber was horizontally divided into 10 segments of equal width and length. The number of propidium iodide stained cells per segment was calculated with Fiji/ImageJ 1.54p. First, the contrast was enhanced by 0.2%, then a “top hat” filter (r = 3 px) was used, followed by binarisation. Lastly, images were binarised and particles between the size of 3–100 px^2^ and with a circularity of ≥0.3 were counted.

### 2.14 Calculation of IC_50_ values

TPZ concentration causing a 50% reduction in cell viability (50% inhibitory concentration; IC_50_) was calculated from the acquired data in Microsoft Excel (Microsoft Corporation, Redmond, WA, United States) using the solver tool with the GRG non-linear algorithm and standard parameters to minimise the sum of squared errors between measured and calculated viabilities using the following formula:

$$y=\min+\frac{\max‐\min}{1+{\left(\frac{x}{{IC}_{50}}\right)}^{Hill\;coefficient}}$$

with x = concentration, y = response (cell viability in %), min = minimum asymptote (plateau) of curve, max = maximum asymptote (plateau) of curve, Hill coefficient = steepness of curve, IC_50_ = concentration causing a 50% reduction of cell viability (half-maximum concentration).

Calculations for 3D samples were executed in the same fashion but from the relative toxicity to control derived from the microscopy data.

### 2.15 Statistical analysis and visualisation

All shown data derived from one single donor, the number of replicates is indicated under each graph. Data are shown as mean value with error bars indicating standard deviation (SD) in positive and negative direction unless indicated otherwise. Data were tested for normal distribution via Shapiro-Wilk test and variance homogeneity was tested with Levene’s test. Statistical significance was assessed with a two-sided Welch’s t-test, applying a Bonferroni correction. Shown asterisks depict the respective Bonferroni-adjusted p-values. Significance was assumed for p-values of 0.05 or smaller. Graphs were generated with Origin Pro® 2025 (OriginLab Corporation, Northampton, MA, United States).

### 2.16 Use of generative artificial intelligence

Generative artificial intelligence (GPT-5.5 Thinking, OpenAI) was used for spelling and grammar correction. All scientific content, data interpretation and phrasing were reviewed and approved by the authors.

## 3 Results

### 3.1 Chamber design and manufacturing

In order to create *in vitro* gradients in 3D hydrogel constructs, which can be easily monitored with a conventional microscope, we developed a simple oxygen gradient chamber which utilises limited oxygen diffusion. The design of the 3D hydrogel oxygen gradient chamber is shown in Figure 1. The chamber consists of a bottom part that serves as a mould for the hydrogel and a top part that enables mechanical fixation of the glass cover above the hydrogel (Figures 1A,B). The top part has an indentation on both sides that fits the 6 mm wide glass slide, clearance holes for the fixation screws and it is secured with M2 screws that screw into the bottom part’s threads. The oxygen gradient chamber was designed for compatibility with conventional cell culture ware and was glued to the bottom of a 6-well plate for its cultivation and subsequent analysis (Figure 1C). The respective. STL files are provided in the Supplementary Material.

**FIGURE 1:**
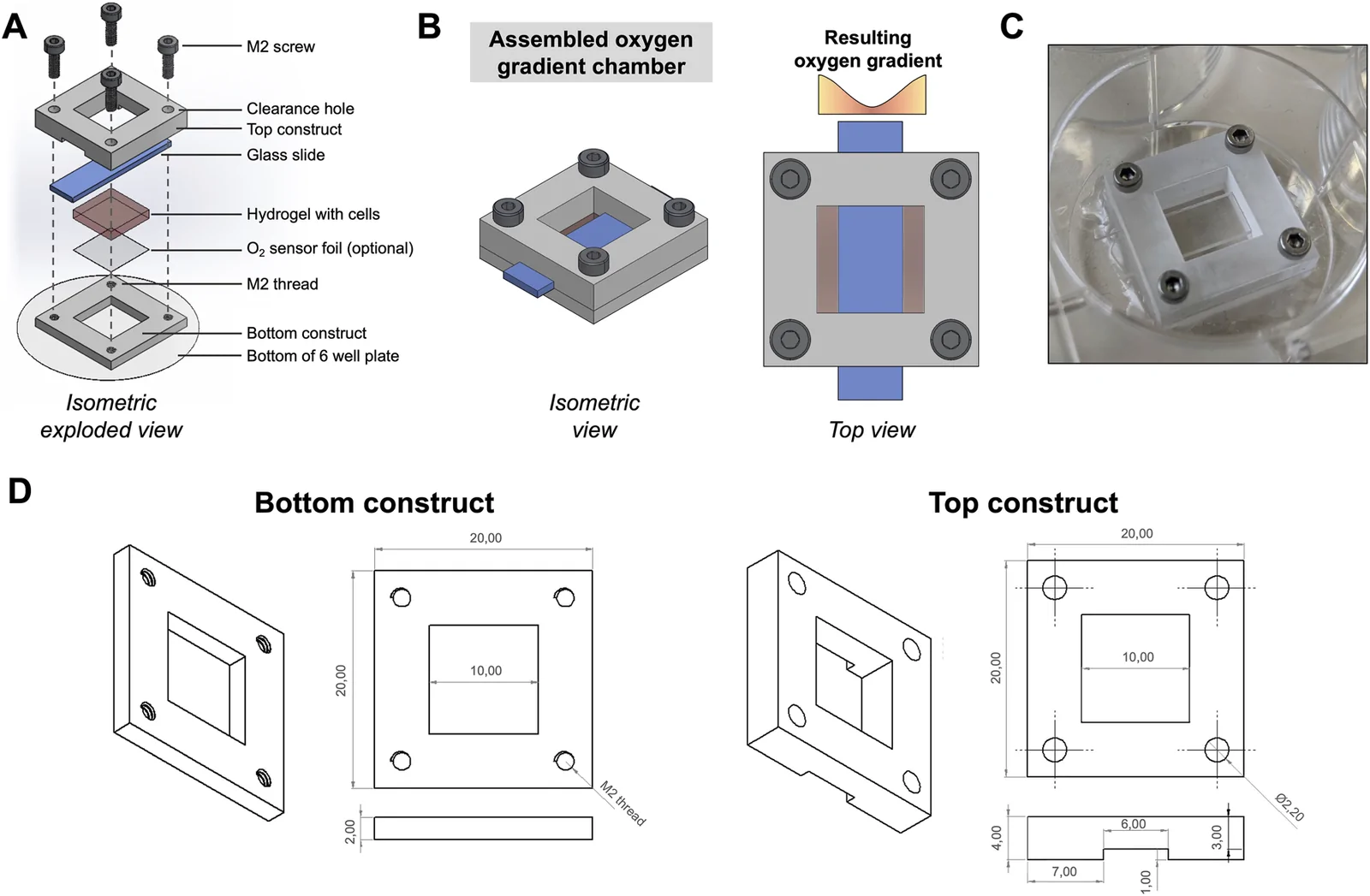
Schematic representation of 3D hydrogel oxygen gradient chamber. **(A)** Isometric exploded view of oxygen gradient chamber. **(B)** Isometric view (left) and top view (right) of assembled oxygen gradient chamber and depiction of expected resulting oxygen gradient inside the hydrogel. **(C)** Photograph of the assembled gradient chamber glued to the bottom of a conventional cell culture 6-well plate. **(D)** Technical drawing of bottom part (left) and top part (right) of the chamber with dimensions presented in mm.

While the bottom construct of the oxygen gradient chamber has a nominal capacity of 200 μL, it was filled with 225 µL to ensure direct contact between the hydrogel surface and the mounted glass slide. The glass covers the central region of the hydrogel and thereby blocks oxygen exchange between the hydrogel and the cell culture medium in which it is submerged. Oxygen exchange is still possible in the non-covered peripheral regions of the hydrogel. This setup was expected to result in the lowest oxygen concentrations where the diffusion distance is the largest, with oxygen concentrations bidirectionally increasing towards the non-covered hydrogel areas (Figure 1B, right).

An oxygen sensor foil can optionally be integrated into the chamber on the bottom to allow spatiotemporally resolved oxygen quantification during cultivation (Figure 1A). The technical drawings in Figure 1D show the exact dimensions of top and bottom parts in mm.

### 3.2 Characterisation of resulting oxygen gradient

To evaluate oxygen gradient formation inside the chamber, non-modified MSCs were embedded in the high DoF GelMA hydrogel and the spatiotemporal oxygen concentration within hydrogel segments after glass mounting was measured using a phosphorescence-based oxygen sensor foil for a time period of over 1 week. Additionally, hypoxia reporter MSCs were embedded in the GelMA hydrogel and UnaG fluorescence reflecting HIF1α stabilization was analysed over time after the glass was mounted on top of the hydrogel.

#### 3.2.1 Visualisation and quantification of oxygen concentrations in gradient chamber segments

To quantify the spatiotemporal changes within the oxygen gradient chamber, oxygen sensor foils were integrated on the bottom of the chamber and the oxygen concentration was monitored over the duration of 8 days (Figure 2A). A non-covered sample (top row) served as a control which was compared to a sample covered with a 6 mm glass slide (bottom row). For subsequent quantification, the full hydrogel area was horizontally segmented within the VisiSens software into a total of 10 segments, as shown in Figure 2C. Segments 3–8 are placed under the glass slide, while segments 1–2 and 9–10 are not covered.

**FIGURE 2:**
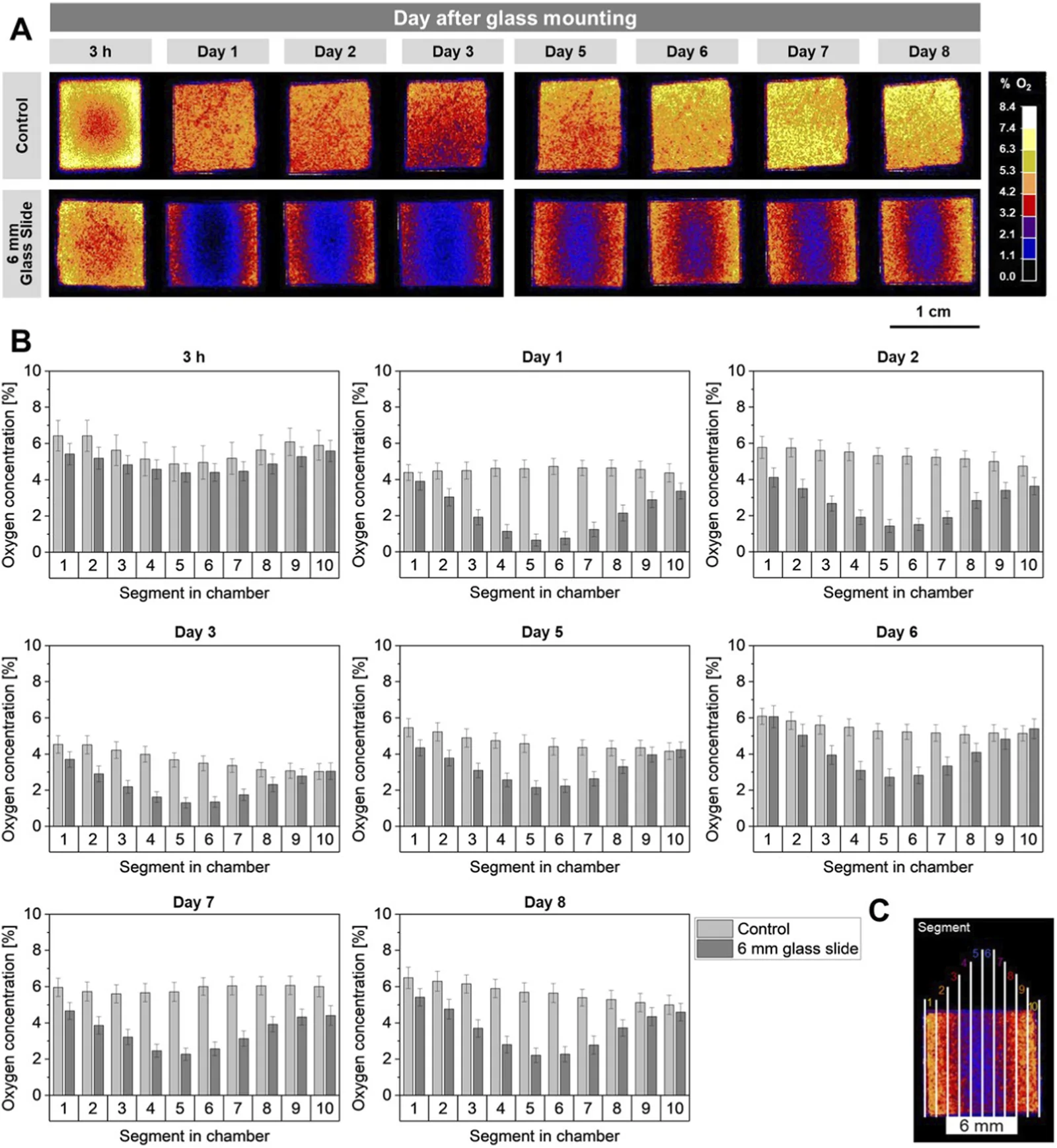
Spatiotemporal changes in the oxygen gradient chamber with high DoF Gelatine methacryloyl hydrogel with and without glass coverage from 3 h until 8 days after glass mounting. **(A)** Spatial distribution of oxygen concentration within one oxygen gradient chamber with and without glass cover. Scale bar represents 1 cm. **(B)** Spatiotemporal quantification of oxygen concentrations in each segment per day. Data are shown as mean ± SD within each segment. **(C)** Schematic representation of segmentation used within the VisiSens software for the quantification of the oxygen concentrations within the oxygen gradient chamber.

In the non-covered control chamber, the oxygen concentration initially showed a slight reduction within the central region 3 h after assembly, with central oxygen values ranging around 5% O_2_ in the middle of the chamber, while outer segments showed a higher concentration of 6%–6.5% O_2_ (Figures 2A,B). The central reduction in O_2_ disappeared in the following days of cultivation. However, a slight decrease in the oxygen concentration from the rear part of the chamber to its front was observed in the control.

In the chamber covered with the 6 mm glass slide, a central reduction of the oxygen concentration was detectable 3 h after glass mounting, and a similar shape of gradient initially also formed in the non-covered sample, indicating the initiation of the formation of a radial bidirectional gradient. After 24 h of glass coverage, a distinct oxygen gradient had formed only in the glass-covered chamber. The lowest O_2_ concentrations were measured in the mid segment underneath the cover with the largest distance to the peripheral non-covered oxygen exchange surface. Oxygen concentration decreased to just under 1% O_2_, while peripheral regions showed a concentration of approximately 3.5%–4% O_2_. Compared to the non-covered control chamber, the glass-covered sample displayed a pronounced oxygen gradient. Even though only segments 3–8 were directly covered by the glass slide, oxygen concentrations in non-covered peripheral segments were lower in the covered chamber at most time points than in the control sample, indicative of a bidirectional gradient formation. After an initial stabilisation phase during the first days of cultivation, where the measured oxygen concentrations were lowest, the oxygen gradient remained relatively stable from day 5 after glass mounting onwards. During this phase of the cultivation, the lowest oxygen concentrations in the central segments ranged around 2% O_2_, while the concentration in the outer segments increased to values between 5%–6% O_2_.

#### 3.2.2 Visualisation of oxygen gradient with the hypoxia reporter cells

To assess the cellular response to the oxygen gradient generated inside the chamber, hypoxia reporter MSCs were embedded in the oxygen gradient chamber and analysed via fluorescence and brightfield microscopy over 10 days after mounting of the glass slide (Figure 3). A distinct UnaG-based hypoxic signal was detected within the central region of the hydrogel underneath the glass cover at 24 h after the cover was mounted, indicative of the formation of a hypoxic region within the hydrogel. Different layers within the hydrogels’ Z-axis did not reveal differences within the Z-axis layers (Supplementary Figure S1). The region with hypoxia reporter signal expanded with continued cultivation, and stabilised at days 4 and 5 after the glass was mounted. Microscopic images covering the complete hydrogel width are shown in Supplementary Figure S2 and are indicative of hydrogel contraction in the outer regions of the hydrogel. Contrasting to the presence of fluorescent hypoxia reporter signal during the first 5 days in low DoF hydrogel, hypoxia reporter signalling was only present for the first 18 h in a high DoF hydrogel with the same setup conditions (Supplementary Figure S3).

**FIGURE 3:**
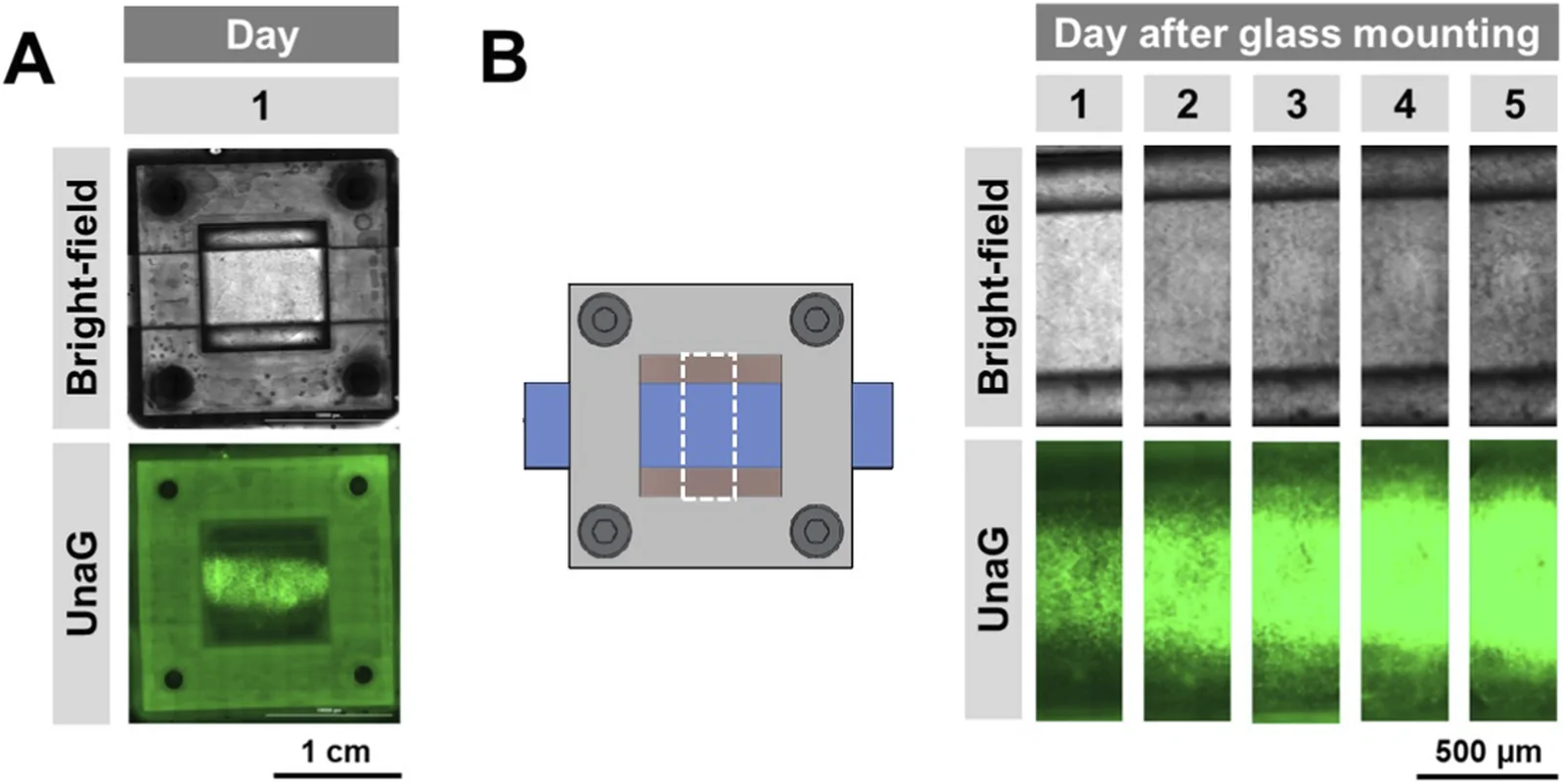
Brightfield (top) and fluorescence (bottom) microscopy of hypoxia reporter MSCs in oxygen gradient chamber at day 1–10 after glass mounting. 7.5% w/v low DoF GelMA seeded with 1 × 10^6^ MSCs per mL of hydrogel. Z-stack projection to single image plane of whole construct thickness acquired with BioTek Cytation 5. **(A)** Microscopy of the whole chamber on day 1 after glass mounting. Scale bar: 1 cm. **(B)** Microscopy of middle section of gradient chamber on day 1–5 after glass mounting. White dotted line in the chamber sketch (left) indicates the depicted microscopy area. Cells are derived from n = 1 donor, and n = 1 technical replicates are shown. Scale bar: 500 µm.

### 3.3 Application of hypoxia-activated prodrug tirapazamine (TPZ)

To investigate whether the generated oxygen gradient can be applied to test spatially dependent drug responses, tirapazamine (TPZ) was used as a hypoxia-activated model prodrug. TPZ cytotoxicity was first evaluated under defined normoxic (21% O_2_) and hypoxic (2.5% O_2_) conditions in 2D culture and 3D GelMA hydrogel discs, before the compound was used within the oxygen gradient chamber.

#### 3.3.1 TPZ toxicity in 2D cell culture

The cytotoxic effect of TPZ was first evaluated in MSCs in a 2D cell culture setting in normoxic and hypoxic conditions. Cells were incubated for 24 h with TPZ concentrations ranging from 6.25–400 µM either at atmospheric oxygen (21%) or hypoxia (2.5%). Cell viability was then assessed with live/dead cell staining as well as with CTB-based quantification of cell viability (Figure 4).

**FIGURE 4:**
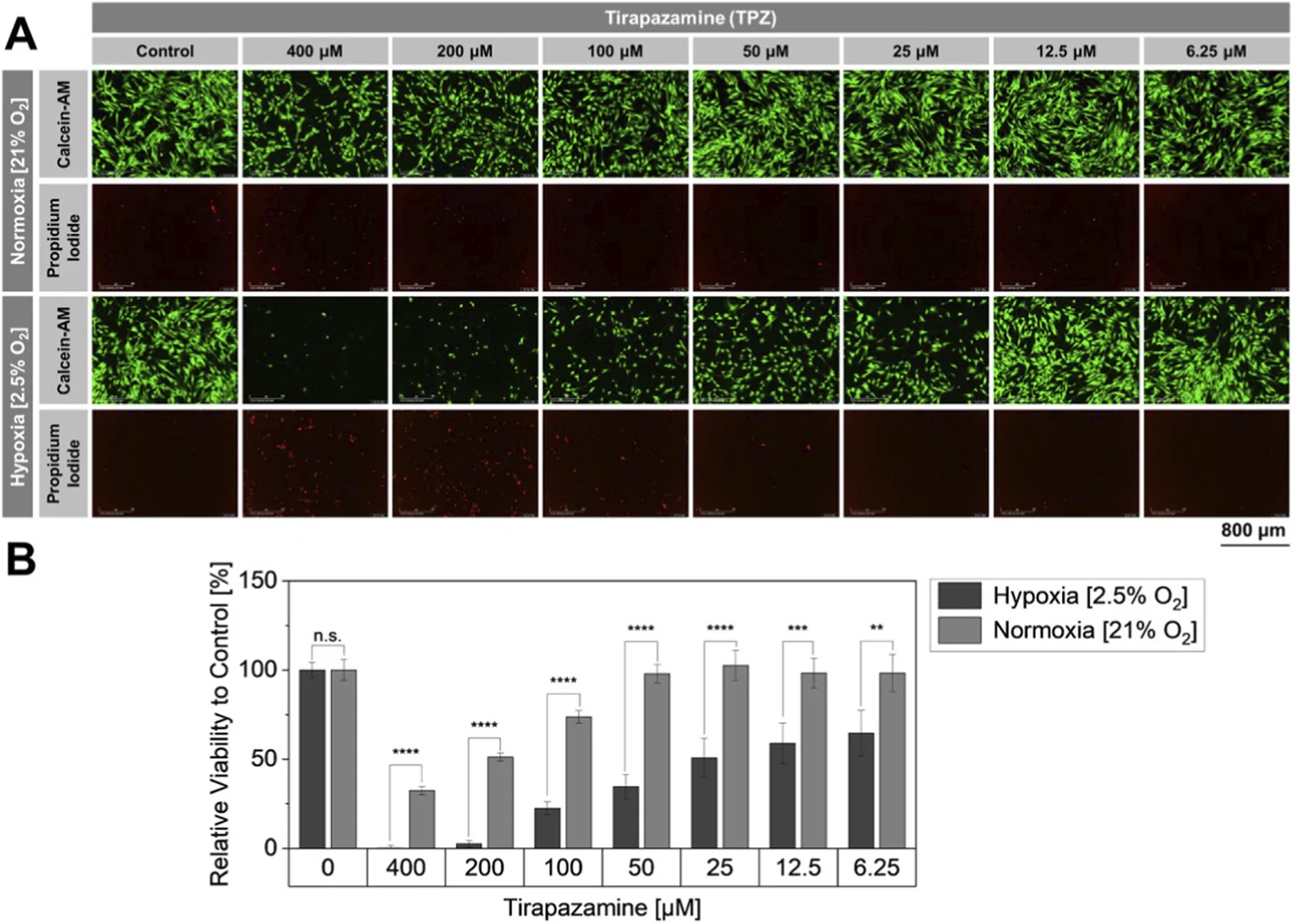
Comparison of tirapazamine (TPZ) toxicity at 0–400 µM in 2D cell culture of MSCs after 24 h of hypoxic (2.5% O_2_) or normoxic (21% O_2_) incubation. **(A)** Fluorescence microscopy of live/dead cell staining with Calcein-AM (green, upper row) and Propidium iodide (red, lower row) after normoxic (21% O_2_, top) and hypoxic (2.5% O_2_, bottom) cultivation. Images acquired with Incucyte S3. Scale bar: 800 µm. **(B)** Quantification of relative viability to control via CTB cell viability assay. Cells are derived from n = 1 donor, and n = 6 technical replicates were analysed. Data are shown as mean ± SD of six replicates. n. s. – not significant, **p < 0.01, ***p < 0.001, ****p < 0.0001.

As shown in Figure 4A, the live/dead cell staining revealed a strong oxygen-dependent increase of TPZ-induced cytotoxicity. The number of viable cells, indicated by Calcein-AM fluorescence, is decreased in the hypoxic samples in a dose-dependent manner. Only a few viable cells with rounded morphology are detectable in the highest TPZ concentrations in the samples cultivated at 2.5% O_2_, while in the normoxic samples only some reduction in confluency was observed. Similarly, the propidium iodide staining confirmed the oxygen-dependent increased TPZ cytotoxicity, with more dead cells in the hypoxia samples than for the corresponding TPZ concentration in normoxia samples.

The quantification of the cell viability using a CTB assay confirmed this observation (Figure 4B). Under normoxic conditions, TPZ concentrations ≤50 µM did not result in a significant reduction in cell viability compared to the mock-treated control. Contrastingly, all TPZ concentrations tested at 2.5% O_2_ resulted in a significantly reduced viability compared to the respective concentration that was cultivated at 21% O_2_ and even the lowest tested TPZ concentration of 6.25 µM reached a relative viability to control of approximately 65%. Thus, the calculated IC_50_ values for MSCs were 148.7 μM at 21% O_2_ and 20.4 μM at 2.5% O_2,_ showing a 7.2-fold increase in cytotoxicity.

#### 3.3.2 TPZ toxicity in 3D hydrogel discs

TPZ toxicity was next investigated in 3D GelMA hydrogel discs in both 7.5% low DoF and 5% high DoF GelMA under normoxic and hypoxic conditions.

The storage modulus G′ of the 7.5% low DoF hydrogel was determined to be 110 Pa, while G′ for the 5% high DoF hydrogel was 1,570 Pa. The degree of functionalisation was determined by TNBS assay to be 29.3% for the low and 99.55% for the high DoF hydrogel, respectively.

Hydrogel discs with encapsulated cells were treated with TPZ concentrations ranging from 6.25–400 µM either for 24 h at atmospheric oxygen levels or at hypoxia (2.5%). Cell viability was subsequently assessed via live/dead cell staining of the whole discs (Figure 5 for high DoF; Supplementary Figure S4 for low DoF). In both GelMA batches, a similar oxygen-dependent TPZ toxicity was observed: Viable cells were still present under normoxic conditions even after treatment with the highest TPZ concentration of 400 μM, showing the strongest Calcein-AM signal around the outer rim of the discs. Contrastingly, hypoxic cultivation at the same concentration resulted in very low viability. Quantification of the images revealed that 400–100 µM TPZ showed a 4.6-times higher cytotoxicity at 2.5% O_2_ compared to atmospheric O_2_ and still a significantly increased cytotoxicity after hypoxic culture when compared to normoxic culture for 50 µM TPZ (Figure 5C). The calculated IC_50_ value at hypoxia was 47.3 µM, while for the normoxic samples, no significant trend in hypoxia-increased cytotoxicity was observed.

**FIGURE 5:**
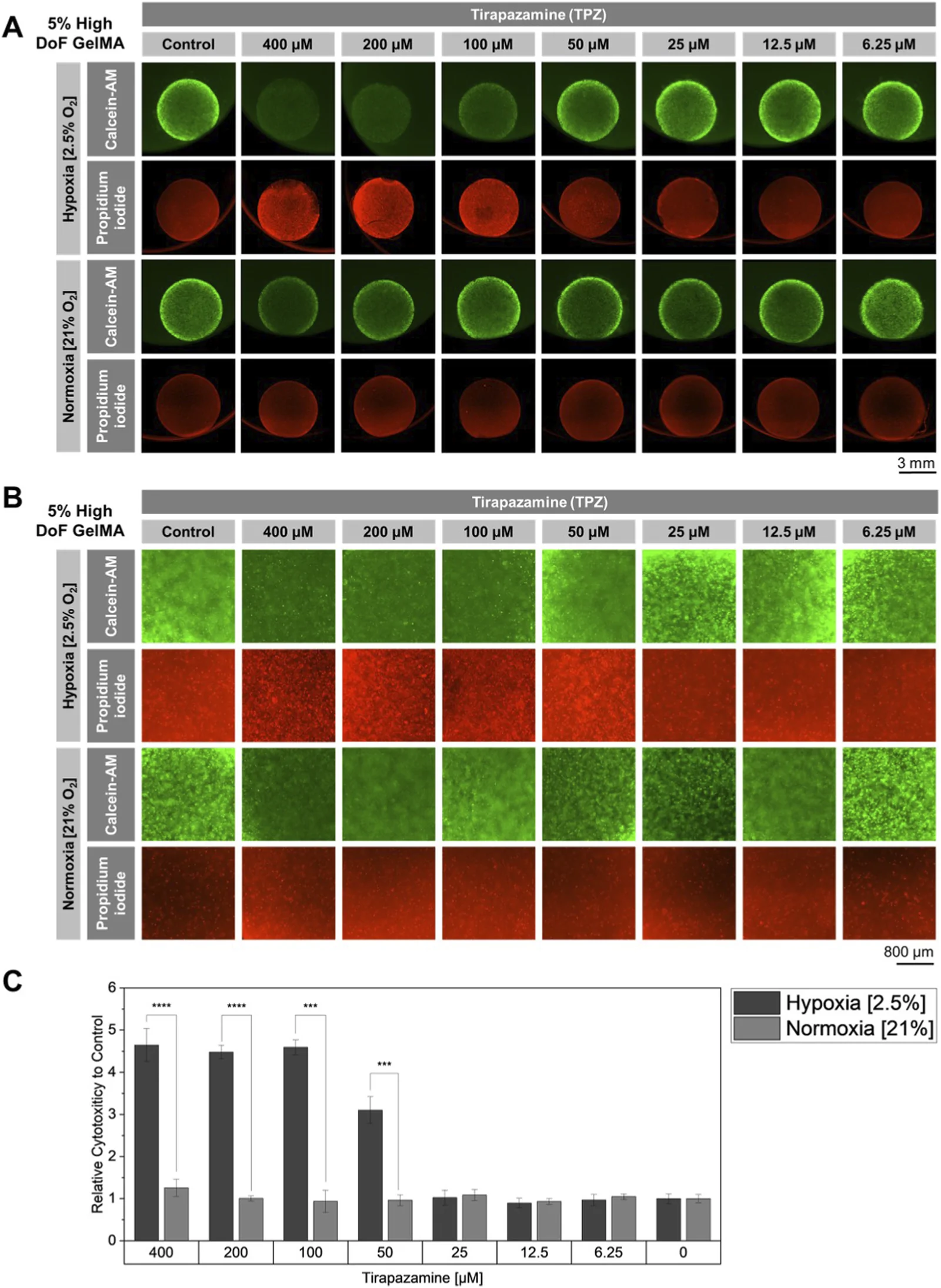
Comparison of tirapazamine (TPZ) toxicity at 0–400 µM in 5% high DoF 3D hydrogel discs seeded with 1 × 10^6^ MSCs per mL of hydrogel after 24 h of hypoxic (2.5% O_2_) or normoxic (21% O_2_) incubation. Fluorescence microscopy of **(A)** full hydrogel construct (Scale bar: 3 mm) and **(B)** Representative magnified view of a selected area (Scale bar: 800 µm) after live/dead cell staining with Calcein-AM (green, upper row) and Propidium iodide (red, lower row) after normoxic (21% O_2_, upper rows) and hypoxic (2.5% O_2_, lower rows) cultivation. One representative image shown of a total of 3 single-plane images acquired with Incucyte S3. **(C)** Quantitative analysis of the relative cytotoxicity to control of TPZ, generated by analysis of PI-stained cells per image. Cells are derived from n = 1 donor, and n = 3 technical replicates were analysed. Data are shown as mean ± SD of three replicates. n. s. – not significant, **p < 0.01, ***p < 0.001, ****p < 0.0001. DoF = Degree of functionalisation, GelMA = Gelatine methacryloyl.

#### 3.3.3 TPZ toxicity in gradient chamber

Lastly, TPZ was applied in oxygen gradient chambers to evaluate whether the created oxygen gradient within the chamber resulted in region- and thus hypoxia-dependent cytotoxicity after 24 h of incubation. Chambers were assembled with or without a 6 mm wide glass cover and therefore an oxygen gradient was created before the addition of 200 µM TPZ. The experimental workflow is shown in Supplementary Figure S5. Cytotoxicity was then assessed with propidium iodide staining (Figure 6). The experimental setup and selected segmentation used during the analysis are shown in Supplementary Figure S6.

**FIGURE 6:**
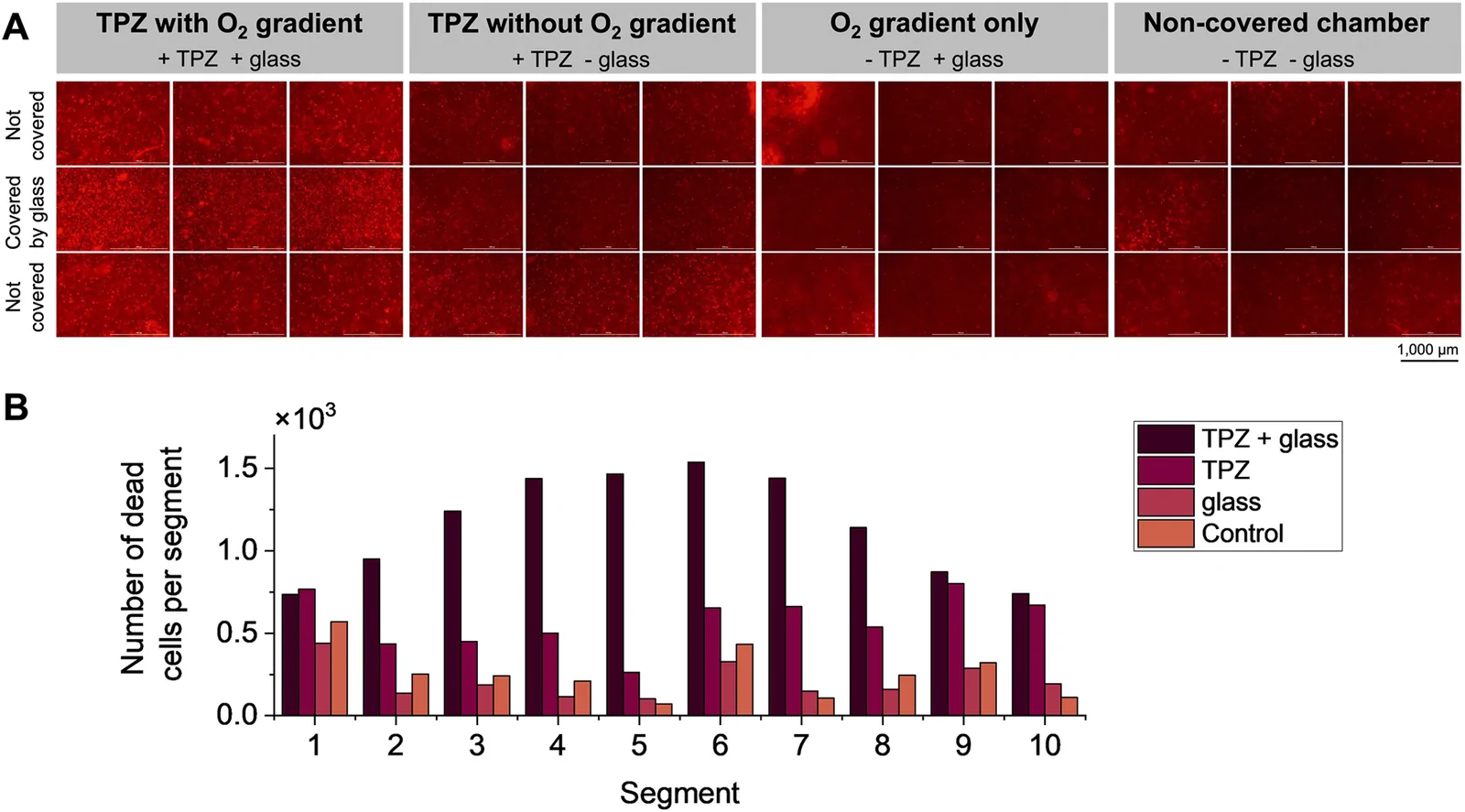
Spatially dependent tirapazamine-induced cytotoxicity in the oxygen gradient chamber (proof-of-concept). **(A)** Propidium iodide stain of oxygen gradient chambers seeded 1 × 10^6^ MSCs per mL of a 5% high DoF hydrogel, cultivated with or without a 6 mm glass cover and treated with or without 200 µM tirapazamine (TPZ) for 24 h. Cells are derived from n = 1 donor, and one chamber per condition was analysed as a proof-of-concept experiment. For each condition, three representative segments of the chamber are shown, corresponding to peripheral (upper and lower row) and central regions (middle row) of the hydrogel. One single plain shown from an acquired Z-stack with BioTek Cytation 5. Scale bar: 1,000 µm. **(B)** Quantification of dead cells per segment. TPZ = Tirapazamine; PI = Propidium iodide; GelMA = Gelatine methacryloyl.

In both TPZ-treated oxygen gradient chambers, an increased number of dead cells was present when compared with the TPZ-free controls (Figures 6A,B). Both TPZ-free controls showed a low number of dead cells, with no increase in the glass-covered area, indicating that the process of covering the hydrogel did not induce substantial cell death during the investigated cultivation period. Images acquired at different focal planes along the hydrogel Z-axis showed a comparable spatial distribution of PI-positive cells without pronounced Z-dependent differences (Supplementary Figure S7). While TPZ treatment resulted in some increased cytotoxicity in the TPZ-treated but non-covered sample, the number of non-viable cells was strongly increased under the glass covered region. This indicates that TPZ-induced cytotoxicity was indeed enhanced in the core hypoxic region of the chamber where oxygen concentration was the lowest.

### 3.4 Further applications of oxygen gradient chamber

To evaluate whether the geometry of the chamber could be adapted to cover a broader area of the hydrogel, an alternative top part for the integration of an 8 mm wide glass slide was designed (Figure 7A). The corresponding. STL file is provided as a Supplementary Material. After 24 h of cultivation with an 8 mm glass slide, hypoxia reporter MSCs embedded in 5% high DoF GelMA showed a distinct and widened UnaG-based hypoxia reporter signal underneath the covered area (Figure 7B) with only few non-viable cells detectable in the propidium iodide stain.

**FIGURE 7:**
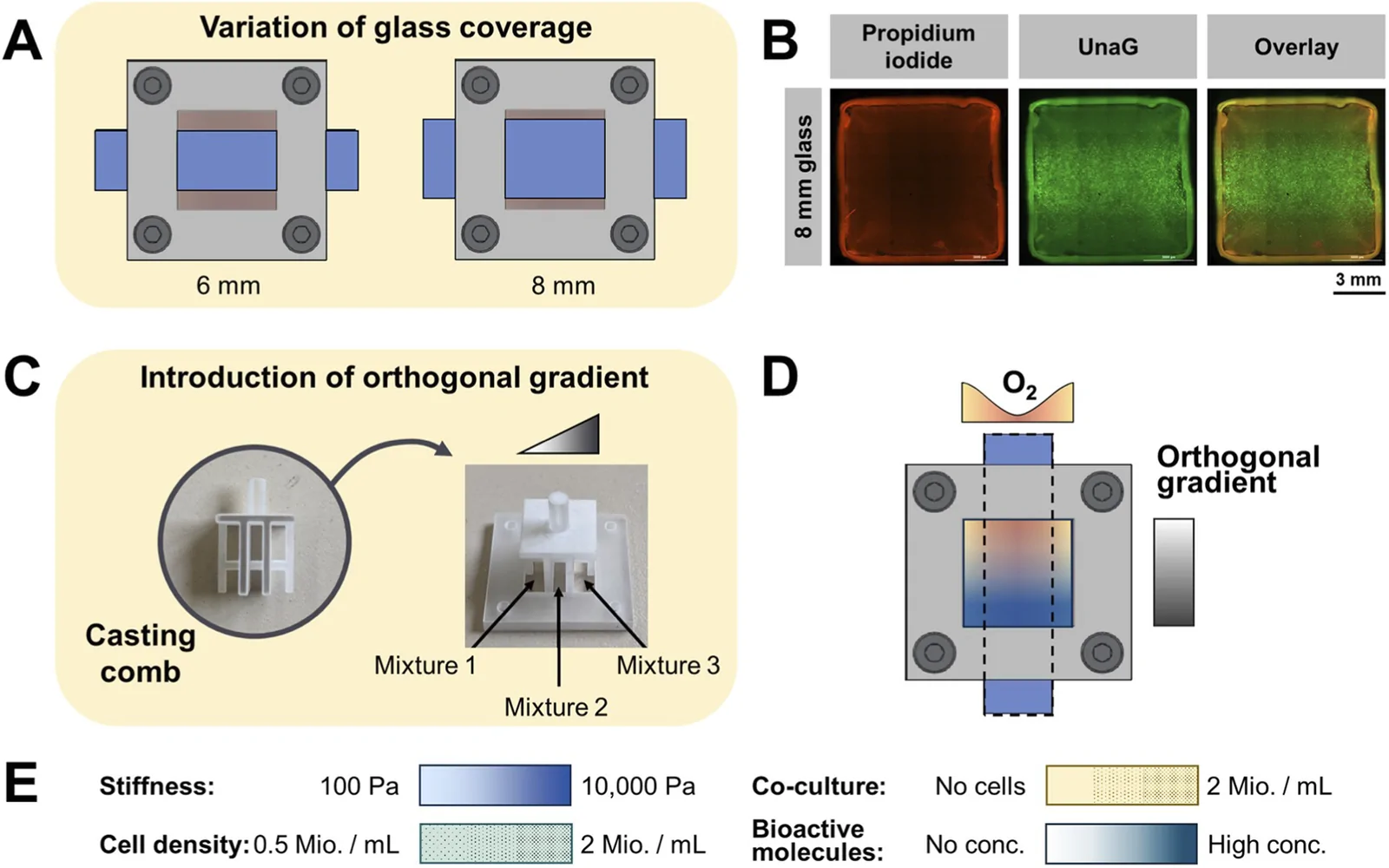
Possible variations of oxygen gradient chamber setup, such as variation of glass width and introduction of orthogonal gradients within hydrogels. **(A)** Schematic comparison of oxygen gradient chamber covered with a 6 mm (left) or 8 mm (right) glass slide. **(B)** Fluorescence microscopy of hypoxia reporter MSCs in 5% high DoF GelMA after 24 h of covering with a glass slide of 8 mm width. Images show dead cell staining with Propidium iodide (red, left) and UnaG-based hypoxia signalling (green, middle) as well as the overlay of dead cell stain and hypoxia signalling (right). Images acquired with BioTek Cytation 5. Scale bar: 3 mm. **(C)** Application of a casting comb during hydrogel pouring, allowing for 3 segments with different hydrogel composition. **(D)** Anticipated shape of oxygen gradient and orthogonal gradient within oxygen gradient chamber. Dotted line marks the area covered by 6 mm glass slide. **(E)** Examples for potential orthogonal gradients. Mio. = Million, Pa = Pascal, conc. = concentration.

Furthermore, a casting comb was designed to enable the generation of three segments of different hydrogel composition during the pouring of the gel in order to create a gradient that is perpendicular to the bidirectional oxygen gradient (Figure 7C). The anticipated shape of the resulting dual gradients is illustrated in Figure 7D. Potential applications include the introduction of a stiffness gradient, a cell seeding density gradient, a gradient with a different seeding density of an additional cell type for co-culture or a gradient of bioactive molecules (Figure 7E).

## 4 Discussion

Given hypoxia’s central role in pathological and physiological processes, relevant *in vitro* models must be developed. Although many different setups have been established and these systems have also contributed to the understanding of oxygen-dependent cell behaviour, most are only applicable to 2D cell culture and a majority also require complex handling procedures in order to prepare or analyse samples and many cannot be monitored with a standard inverted microscope. This can limit their implementation into a standard cell culture laboratory.

The aim of the present study was to develop and characterise a simple and low-cost 3D hydrogel-based oxygen gradient chamber that can be manufactured by 3D printing and used within conventional cell culture ware. The chambers rely on the partial coverage of a cell-laden hydrogel with a glass slide, which locally restricts the O_2_ exchange between the hydrogel and the surrounding cell culture medium, while oxygen exchange remains possible in the non-covered, peripheral regions. The cellular oxygen consumption contributes to lowered oxygen levels underneath the covered central region. The oxygen gradient chambers form a spatial oxygen gradient without the need for gas perfusion, external pumps or complex chamber or microfluidic geometries. Since the chamber can be integrated into a standard 6-well plate and analysed with conventional microscopy systems, it provides increased accessibility for the targeted generation of oxygen gradients in 3D hydrogel cultures.

Direct oxygen measurements using integrated oxygen sensor foils confirmed the presence of a distinct, spatially defined oxygen gradient. In non-covered control chambers, only a slight and transient gradient was detected during the early cultivation stage in its central region. Contrastingly, glass-covered chambers showed a decrease in oxygen concentration underneath the covered area. The higher variability of oxygen concentrations observed at the hydrogels rim may be related to changes in hydrogel geometry. Although the hydrogels were designed to fit flush with the chamber, some lateral access of medium between the hydrogel and the bottom construct cannot be excluded, which would increase the effective diffusion surface and could contribute to the observed edge effects. Furthermore, there could be a possible contribution of the printing material, which does show some autofluorescence and could have influenced the measurements with the sensor foils, or used adhesives that were both not directly assessed. Following an initial stabilisation period where oxygen levels in the central regions transiently dropped to just under 1% and peripheral regions measured approximately 4%, the gradient remained relatively stable in its shape and the measured spatial oxygen values from day 5 onwards. The increase of oxygen levels after the initial low-oxygen phase may be due to changes in cellular adaptation to the microenvironment and should be considered when planning the experimental setup and time frames. The central oxygen value stabilised afterwards at 2% O_2_ in the central region underneath the glass slide and varied between 5%–6% in the peripheral region. The measured oxygen levels within the gradient are known to induce HIF-dependent response in many cell types while still facilitating cell survival (Malda et al., 2007; Pattappa et al., 2019; Schmitz et al., 2020; Volkmer et al., 2008). A bidirectional oxygen gradient over several days was maintained without the need of active external manipulation. Though the oxygen sensor foils were used as a crucial validation approach, they could not be implemented in all experiments due to their lack of translucency. The integration of a different oxygen measurement method, such as commercially available oxygen sensor beads, could circumvent this limitation in future experiments.

In addition to direct oxygen measurements, hypoxia reporter MSCs were used to evaluate the biological response to the generated oxygen gradient. These reporter cells have previously been established as a tool for monitoring cellular hypoxia in 2D and 3D culture systems (Schmitz et al., 2020; 2022). This reporter system is based on HIF-1-dependent expression of the fluorescent protein UnaG. Previous studies demonstrated that UnaG fluorescence negatively correlates with oxygen concentration, providing a quantitative readout (Dienemann et al., 2023; 2025; Schmitz et al., 2020). Hypoxia reporter MSCs seeded inside the oxygen gradient chamber showed a distinct fluorescence signal in the central hydrogel region already 24 h after glass mounting, corresponding to the area with the largest diffusion distance to the non-covered hydrogel areas. The hypoxia reporter signal expanded with increasing cultivation time until it stabilised at day 4 and day 5. Oxygen profiles in 3D cell culture constructs cannot only be influenced by diffusion, but also by cellular proliferation and oxygen consumption adjustment, matrix remodelling or hydrogel contraction/swelling throughout its cultivation (Carroll et al., 2021; Colom et al., 2014; Fiedler et al., 2014; Malda et al., 2007; Schmitz et al., 2022; Volkmer et al., 2008; Wagner et al., 2011). Hydrogel contraction, as observed in the peripheral areas of the chamber, may alter the geometry in such a way that it changes diffusion area over time and thus needs to be kept at a minimum for best reproducibility of results.

Interestingly, reporter MSCs encapsulated in high DoF GelMA showed hypoxia reporter signalling only during the initial period after glass mounting. This difference between the cellular reactions to low and high DoF GelMA may be related to differences in hydrogel stiffness, pore size, cell-mediated contraction and the resulting cellular activity. The reduction in cellular reporter signal intensity in stiffer hydrogels is consistent with the findings of Schmitz *et al.* who demonstrated that hypoxia signalling in GelMA-based 3D cultures of higher stiffnesses is not mainly reduced due to lower hydrogel oxygen permeability, but is mediated by cellular adaptations to hydrogels with higher storage moduli (Schmitz et al., 2022). However, the present study did not aim to systematically investigate the mechanisms underlying the different reporter responses in low and high DoF GelMA. While the hypoxia reporter signal does not reproduce the initial oxygen minimum detected by the direct oxygen measurement, this does not necessarily represent a contradiction between the two readouts. The early oxygen minimum detected by the sensor foils may indicate the initial establishment of the gradient underneath the glass-covered region, but the fluorescent protein accumulation within the cells is delayed by several hours after transfer of cells to the hypoxic environment. While Schmitz *et al.* showed some increase in hypoxia reporter signal during the first 10 h after hypoxic incubation at 2.5%, the highest signal intensity was detected 24 h after transfer to hypoxic culture (Schmitz et al., 2020). Thus, the differences between these measurements may be due to the temporal offset between the introduction of oxygen limitation and the cellular response, which should be further investigated in future studies.

In this study, cells isolated from one single donor were used and present a limitation of this work, which may restrict the generalisability of the cellular response in the gradient, as well as the gradient profile itself. Moreover, since, different cell types have different oxygen consumption rates and metabolic activity, any gradient profile created with the presented system must be characterised for each cell type intended to be used in future experiments. An overall limitation of this used gradient characterisation set-up with oxygen sensor foils and hypoxia reporter cells is that the oxygen gradient across the hydrogel thickness is not quantitatively resolved in Z-axis direction. Oxygen was quantitatively measured only at the hydrogel-foil interface at the construct bottom, whereas the hypoxia reporter cell signalling Z-stacks provide qualitative information the hypoxic regions within the constructs. Thus, while our data support the formation of 3D oxygen gradient, the exact shape of the O_2_ profile along the Z-axis would benefit from the future determination using advanced microsensor techniques or computational modelling.

As a proof-of-concept, the hypoxia-activated prodrug tirapazamine (TPZ) was used to investigate whether the generated oxygen gradient would also be able to induce a spatially dependent increase in cytotoxicity. TPZ is a bio-reductive compound with increased cytotoxicity at low oxygen tensions in which radical TPZ-derived intermediates cannot be reoxidised due to the lack of oxygen, thus inducing DNA damage (Brown, 1993; Denny and Wilson, 2000; Peters and Brown, 2002). Although clinical translation of TPZ has been very limited, this compound remains a useful model substance for evaluating oxygen-dependent cytotoxicity changes *in vitro* (Rischin et al., 2010; Spiegelberg et al., 2019; Von Pawel et al., 2000).

In 2D MSC culture, TPZ showed a significant increase in hypoxia-induced cytotoxicity. The calculated IC_50_ value decreased from 148.7 µM under atmospheric oxygen conditions to 20.4 μM at 2.5% O_2_. Published TPZ toxicity data at normoxia *versus* hypoxia show that IC_50_ values generally show higher TPZ potency under low oxygen tensions than under normoxia (Guise et al., 2020; Patterson et al., 1995; 1997; Saunders et al., 2000). The IC_50_ values from this study are most comparable to those reported by Blaszczak-Swiatkiewicz *et al.*, who measured TPZ-induced cytotoxicity in A549 lung adenocarcinoma cells after 48 h of incubation that resulted in an IC_50_ of 166.2 μM at atmospheric O_2_ and 30 μM at 1% O_2_ with a 4.61-fold increased cytotoxicity in hypoxia (Blaszczak-Swiatkiewicz et al., 2013). IC_50_ values from literature vary extensively as different concentrations of oxygen (most often even anoxia), cells and incubation times were used. Consequently, increase in hypoxia-dependent cytotoxicity also varied considerably between a 2-fold–100-fold increase (Alimohammadvand et al., 2025; Blaszczak-Swiatkiewicz et al., 2013; Koch, 1993; Patterson et al., 1995; 1997; Saunders et al., 2000).

Hypoxia-associated TPZ cytotoxicity was also present in 3D GelMA hydrogel discs. In both low and high DoF GelMA discs, incubation with 400 to 100 µM TPZ at 2.5% O_2_ resulted in a significant reduction of viable cells, as quantified by live/dead staining. More viable cells were consistently observed at disc peripheries, likely due to central oxygen depletion due to diffusion limitations. This is similar to findings by Schmitz *et al.* in their study on thick hydrogel discs with hypoxia reporter cells (Schmitz et al., 2022). To minimise diffusion effects in future investigations, hydrogel disks could be miniaturised and/or the spatial distribution of oxygen within the discs could be additionally monitored. While TPZ toxicity has been extensively studied in 2D cell cultures, spheroids or directly *in vivo* (Close and Johnston, 2022; Evans et al., 2020; Wan et al., 2016), only few publications investigated the use of TPZ in scaffold-based 3D cell culture settings (Lu-Chinh Phan et al., 2020; Ornell et al., 2019). To our knowledge, the effect of TPZ in hypoxia *versus* normoxia has been compared only once in a silk-scaffold approach with neuroblastoma cells with no direct reporting of IC_50_ values (Ornell et al., 2019). While IC_50_ values have been reported for Matrigel-based hydrogels with TPZ without additional oxygen control during the culture (Lu-Chinh Phan et al., 2020), the current number of publications is very limited. This present study estimated an IC_50_ value for MSCs at atmospheric oxygen levels of 148.7 µM in 2D vs. >400 µM in 3D hydrogel discs, and of 20.4 µM in 2D vs. 47.3 µM in 3D hydrogel discs at hypoxia (2.5% O_2_), indicating that substantially higher TPZ concentrations are required to achieve equivalent cytotoxicity in 3D GelMA hydrogel cultures and thus emphasising the importance of selecting an appropriate culture model for drug response testing.

Lastly, TPZ was applied within the oxygen gradient chamber to test whether the created oxygen gradient also translated into a spatially defined cytotoxic response. Oxygen gradients were generated by mounting of glass slides for 24 h before the addition of 200 µM TPZ for 24 h. TPZ-treated samples showed an overall increased number of dead cells compared with untreated controls, but only the glass-covered sample showed an additional increase in the central region of the sample where the oxygen limitation is most pronounced. Thus, the oxygen gradient chamber converted a physical oxygen gradient into a biologically and pharmacologically relevant readout. Though the spatial difference in TPZ-induced cell death was clearly detectable and in agreement with the oxygen gradient’s shape, the non-covered regions of the chamber also showed lower oxygen concentrations that induced residual TPZ toxicity, likely due to moderate hypoxia (4%–6% O_2_). Although our experimental design was aiming to equalise TPZ exposure and accord for potential diffusion limitations created by glass coverage, we cannot fully exclude differences in the local drug concentration in the presence or absence of the glass cover. However, the spatial pattern of cytotoxicity supports the conclusion that the observed effect in primarily driven by oxygen-dependent TPZ activation rather than by differences in drug distribution alone. Generally, it cannot be excluded that diffusion limitations of TPZ played an additional role and the different assessment of local TPZ concentrations should be used in future studies to elucidate the extent of additional diffusion limitations.

GelMA hydrogel was used in this study because of its high transparency, good biocompatibility and mechanical tunability, making it an excellent candidate for microscopy-based 3D culture experiments (Chai et al., 2026; Pepelanova et al., 2018; Shirahama et al., 2016). However, the presented chamber is not limited to GelMA only and could be used with different hydrogels, which is an advantage for tissue engineering and disease modelling applications. One limiting factor for the matrix of choice is that hydrogel swelling must not be excessive in order to enable subsequent glass mounting without too much additional pressure. Another limitation of this study is the potential additional influence of the hydrogel stiffness and potential cellular reaction due the compression in samples with mounted glass plates on top. Even though we did not have any indication for glass-induced cytotoxicity, or changes in the cells’ reaction to different mechanotransduction, during the adaptation of the oxygen gradient chamber to different hydrogels and/or cell types, seeding densities and hydrogel composition should therefore be optimised to meet the demands of the specific experiment. Future investigations could also include the integration of orthogonal gradients that could be of interest for, but are not necessarily limited to, cell migration experiments, co-culture effects and testing of concentration gradients of bioactive or pharmacological molecules.

The oxygen gradients presented in this work are established by diffusion and defined by the chamber geometry, presence of glass covers as well as by metabolic activity of the embedded cells. Although, based on this design we expect the gradients be stable over the time ranges used in our experiments, longer cultivation periods can lead to increased cell density and changes in metabolic demand, leading to changes of local oxygen concentrations. Moreover, batch-to-batch variability of hydrogels and media exchange may introduce differences in absolute oxygen concentrations and gradient profile. Future studies will benefit from systematic quantification of gradient stability over time under different cell densities, used hydrogel materials and medium-change schedules. Moreover, the mechanism underlying the observed reporter activity after 18 h of cultivation in high-DoF GelMA requires further investigation.

Taken together, the present study demonstrates a simple and accessible 3D hydrogel-based oxygen gradient chamber. The system generates spatial oxygen gradients through the combined effect of a diffusion barrier with oxygen depletion caused by cellular metabolism. It does not require any pumps, gas channels or gas-permeable membranes, introduction of oxygen scavengers, complex microfluidics or high-resolution 3D printing. Oxygen gradient formation was demonstrated by hypoxia reporter signalling of MSCs and directly quantified using oxygen sensor foils. As a functional proof of concept, the hypoxia-activated prodrug TPZ induced enhanced cell death in the hypoxic region of the chamber, while also showing a hypoxia-dependent increase in cytotoxicity in 2D cell culture and 3D hydrogel discs. This oxygen gradient chamber offers a versatile platform for studying hypoxia responses and oxygen-dependent drug effects in a 3D environment. Its simple geometry and compatibility with standard cell culture ware may enable a broader implementation of oxygen gradient experiments in biomaterial research, tissue engineering and disease modelling.

## Data Availability

The .STL files required to fabricate the chamber are provided as Supplementary Material. Additional raw data supporting the findings of this study are available from the corresponding author upon reasonable request.

## References

[B1] Al Tameemi W. Dale T. P. Al-Jumaily R. M. K. Forsyth N. R. (2019). Hypoxia-modified cancer cell metabolism. Front. Cell Dev. Biol. 7, 4. 10.3389/FCELL.2019.00004/BIBTEX 30761299 PMC6362613

[B2] Alimohammadvand S. Shahpouri M. Adili Aghdam M. A. Majdi H. Hamishehkar H. Kaveh Zenjanab M. (2025). Tumor microenvironment responsive TPZ-loaded core-shell polymeric nanoparticles for selective cancer bioreductive therapy. Adv. Pharm. Bull. 15, 390–405. 10.34172/apb.025.43945 40922751 PMC12413963

[B3] Allen J. W. Bhatia S. N. (2003). Formation of steady-state oxygen gradients *in vitro*:application to liver zonation. Biotechnol. Bioeng. 82, 253–262. 10.1002/bit.10569 12599251

[B4] Ando Y. Ta H. P. Yen D. P. Lee S. S. Raola S. Shen K. (2017). A microdevice platform recapitulating hypoxic tumor microenvironments. Sci. Rep. 7 (17), 15233. 10.1038/s41598-017-15583-3 29123197 PMC5680268

[B5] Blaszczak-Swiatkiewicz K. Almeida D. C. De Jesus Perry M. Mikiciuk-Olasik E. (2013). Synthesis, anticancer activity and UPLC analysis of the stability of some new Benzimidazole-4,7-dione derivatives. Molecules 19, 400–413. 10.3390/MOLECULES19010400 24384922 PMC6271476

[B6] Blatchley M. R. Hall F. Ntekoumes D. Cho H. Kailash V. Vazquez-Duhalt R. (2021). Discretizing three‐dimensional oxygen gradients to modulate and investigate cellular processes. Adv. Sci. 8, 2100190. 10.1002/ADVS.202100190 34151527 PMC8292886

[B7] Boyce M. W. Simke W. C. Kenney R. M. Lockett M. R. (2019). Generating linear oxygen gradients across 3D cell cultures with block-layered oxygen controlled chips (BLOCCs). Anal. Methods 12, 18–24. 10.1039/c9ay01690b 32190125 PMC7079814

[B8] Brown J. M. (1993). SR 4233 (tirapazamine): a new anticancer drug exploiting hypoxia in solid tumours. Br. J. Cancer 67, 1163–1170. 10.1038/BJC.1993.220 8512801 PMC1968495

[B9] Carreau A. Hafny Rahbi B. E. Matejuk A. Grillon C. Kieda C. (2011). Why is the partial oxygen pressure of human tissues a crucial parameter? Small molecules and hypoxia. J. Cell. Mol. Med. 15, 1239–1253. 10.1111/j.1582-4934.2011.01258.x 21251211 PMC4373326

[B10] Carroll S. F. Buckley C. T. Kelly D. J. (2021). Measuring and modeling oxygen transport and consumption in 3D hydrogels containing chondrocytes and stem cells of different tissue origins. Front. Bioeng. Biotechnol. 9, 591126. 10.3389/fbioe.2021.591126 34124013 PMC8188180

[B11] Catoira M. C. Fusaro L. Di Francesco D. Ramella M. Boccafoschi F. (2019). Overview of natural hydrogels for regenerative medicine applications. J. Mater. Sci. Mater. Med. 30, 1–10. 10.1007/s10856-019-6318-7 31599365 PMC6787111

[B12] Chai M. He Q. Yao B. Wei R. (2026). Transparent GelMA biomaterials: advanced solutions for ocular tissue engineering and regenerative ophthalmology. Adv. Ophthalmol. Pract. Res. 6, 103–115. 10.1016/J.AOPR.2026.01.001 41909588 PMC13019815

[B13] Chang C. W. Cheng Y. J. Tu M. Chen Y. H. Peng C. C. Liao W. H. (2014). A polydimethylsiloxane–polycarbonate hybrid microfluidic device capable of generating perpendicular chemical and oxygen gradients for cell culture studies. Lab. Chip 14, 3762–3772. 10.1039/C4LC00732H 25096368

[B14] Cheema U. Brown R. A. Alp B. MacRobert A. J. (2008). Spatially defined oxygen gradients and vascular endothelial growth factor expression in an engineered 3D cell model. Cell. Mol. Life Sci. 65, 177–186. 10.1007/S00018-007-7356-8/METRICS 17994289 PMC11131842

[B15] Chen Y. A. King A. D. Shih H. C. Peng C. C. Wu C. Y. Liao W. H. (2011). Generation of oxygen gradients in microfluidic devices for cell culture using spatially confined chemical reactions. Lab. Chip 11, 3626–3633. 10.1039/c1lc20325h 21915399

[B16] Close D. A. Johnston P. A. (2022). Detection and impact of hypoxic regions in multicellular tumor spheroid cultures formed by head and neck squamous cell carcinoma cells lines. SLAS Discov. 27, 39–54. 10.1016/J.SLASD.2021.10.008 35058175

[B17] Colom A. Galgoczy R. Almendros I. Xaubet A. Farré R. Alcaraz J. (2014). Oxygen diffusion and consumption in extracellular matrix gels: implications for designing three-dimensional cultures. J. Biomed. Mater. Res. A 102, 2776–2784. 10.1002/JBM.A.34946 24027235

[B18] Denny W. A. Wilson W. R. (2000). Tirapazamine: a bioreductive anticancer drug that exploits tumour hypoxia. Expert Opin. Investig. Drugs 9, 2889–2901. 10.1517/13543784.9.12.2889 11093359

[B19] Dienemann S. Schmidt V. Fleischhammer T. Mueller J. H. Lavrentieva A. (2023). Comparative analysis of hypoxic response of human microvascular and umbilical vein endothelial cells in 2D and 3D cell culture systems. J. Cell. Physiol. 238, 1111–1120. 10.1002/jcp.31002 36947660

[B20] Dienemann S. Wohlenberg O. J. Gerstenberger J. G. Lavrentieva A. Pepelanova I. (2025). 3D culture of neural progenitor cells in gelatin norbornene (GelNB) hydrogels: mechanical tuning and hypoxia characterization. Front. Bioeng. Biotechnol. 13, 1579580. 10.3389/fbioe.2025.1579580 40521088 PMC12163324

[B21] Dominici M. Le Blanc K. Mueller I. Slaper-Cortenbach I. Marini F. C. Krause D. S. (2006). Minimal criteria for defining multipotent mesenchymal stromal cells. The international society for cellular therapy position statement. Cytotherapy 8, 315–317. 10.1080/14653240600855905 16923606

[B22] Erapaneedi R. Belousov V. V. Schäfers M. Kiefer F. (2016). A novel family of fluorescent hypoxia sensors reveal strong heterogeneity in tumor hypoxia at the cellular level. EMBO J. 35, 102–113. 10.15252/embj.201592775 26598532 PMC4718004

[B23] Evans M. A. Wyatt Shields C. I. Krishnan V. Li-Wen Wang L. Zhao Z. Ukidve A. (2020). Macrophage-mediated delivery of hypoxia-activated prodrug nanoparticles. Adv. Ther. (Weinh). 3, 1900162. 10.1002/ADTP.201900162

[B24] Fiedler T. Belova I. V. Murch G. E. Poologasundarampillai G. Jones J. R. Roether J. A. (2014). A comparative study of oxygen diffusion in tissue engineering scaffolds. J. Mater. Sci. Mater. Med. 25, 2573–2578. 10.1007/S10856-014-5264-7 25016936

[B25] Fleischhammer T. M. Dienemann S. Ulber N. Pepelanova I. Lavrentieva A. (2024). “Detection of hypoxia in 2D and 3D cell culture systems using genetically encoded fluorescent hypoxia sensors,” in Hypoxia: Methods and Protocols. Editor Gilkes D. M. (New York, NY: Humana), 31–48. 10.1007/978-1-0716-3633-6_2 38319567

[B26] Funamoto K. Zervantonakis I. K. Liu Y. Ochs C. J. Kim C. Kamm R. D. (2012). A novel microfluidic platform for high-resolution imaging of a three-dimensional cell culture under a controlled hypoxic environment. Lab. Chip 12, 4855–4863. 10.1039/C2LC40306D 23023115 PMC4086303

[B27] Guise C. P. Abbattista M. R. Anderson R. F. Li D. Taghipouran R. Tsai A. (2020). Subcellular location of tirapazamine reduction dramatically affects aerobic but not anoxic cytotoxicity. Molecules 25 4888. 10.3390/MOLECULES25214888 33105798 PMC7660101

[B28] Höckel M. Vaupel P. (2001). Tumor hypoxia: definitions and current clinical, biologic, and molecular aspects. J. Natl. Cancer Inst. 93, 266–276. 10.1093/jnci/93.4.266 11181773

[B29] Hsu H. H. Ko P. L. Peng C. C. Cheng Y. J. Wu H. M. Tung Y. C. (2023). Studying sprouting angiogenesis under combination of oxygen gradients and co-culture of fibroblasts using microfluidic cell culture model. Mater. Today Bio 21, 100703. 10.1016/J.MTBIO.2023.100703 37483382 PMC10359940

[B30] Ivanovic Z. (2009). Hypoxia or *in situ* normoxia: the stem cell paradigm. J. Cell. Physiol. 219, 271–275. 10.1002/jcp.21690 19160417

[B31] Kapałczyńska M. Kolenda T. Przybyła W. Zajączkowska M. Teresiak A. Filas V. (2018). 2D and 3D cell cultures – a comparison of different types of cancer cell cultures. Archives Med. Sci. 14, 910–919. 10.5114/aoms.2016.63743 30002710 PMC6040128

[B32] Keith B. Simon M. C. (2007). Hypoxia-inducible factors, stem cells, and cancer. Cell 129, 465–472. 10.1016/j.cell.2007.04.019 17482542 PMC3150586

[B33] Koch C. J. (1993). Unusual oxygen concentration dependence of toxicity of SR-4233, a hypoxic cell toxin. Cancer Res. 53, 3992–3997. Available online at: http://www.ncbi.nlm.nih.gov/pubmed/8358728 (Accessed May 8, 2026). 8358728

[B34] Koens R. Tabata Y. Serrano J. C. Aratake S. Yoshino D. Kamm R. D. (2020). Microfluidic platform for three-dimensional cell culture under spatiotemporal heterogeneity of oxygen tension. Apl. Bioeng. 4, 016106. 10.1063/1.5127069 32161836 PMC7060087

[B35] Lu-Chinh Phan N. Pham K. D. Thi-Thanh Nguyen M. Kim N. P. Truong K. D. Van Pham P. (2020). Anti-tumor activity of plant extracts against human breast cancer cells are different in monolayer and three-dimensional cell culture screening models: a comparison on 34 extracts. Biomed. Res. Ther. 7, 3667–3677. 10.15419/bmrat.v7i3.593

[B36] Malda J. Klein T. J. Upton Z. (2007). The roles of hypoxia in the *in vitro* engineering of tissues. Tissue Eng. 13, 2153–2162. 10.1089/ten.2006.0417 17516855

[B37] Malda J. Visser J. Melchels F. P. Jüngst T. Hennink W. E. Dhert W. J. A. (2013). 25th anniversary article: engineering hydrogels for biofabrication. Adv. Mater. 25, 5011–5028. 10.1002/adma.201302042 24038336

[B38] Orabi M. Duan K. Zhou M. Lo J. F. (2025). Microfluidic oxygen gradient assay unveils metabolic shifts in HaCaT cell migration under diabetic conditions. Integr. Biol. 17, zyaf006. 10.1093/INTBIO/ZYAF006 40329922

[B39] Ornell K. J. Mistretta K. S. Newman E. Ralston C. Q. Coburn J. M. (2019). Three-dimensional, scaffolded tumor model to study cell-driven microenvironment effects and therapeutic responses. ACS Biomater. Sci. Eng. 5, 6742–6754. 10.1021/acsbiomaterials.9b01267 33423492

[B40] Palacio-Castañeda V. Kooijman L. Venzac B. Verdurmen W. P. R. Gac S. L. (2020). Metabolic switching of tumor cells under hypoxic conditions in a tumor-on-a-chip model. Micromachines (Basel) 11, 382. 10.3390/MI11040382 32260396 PMC7231186

[B41] Palacio-Castañeda V. Velthuijs N. Le Gac S. Verdurmen W. P. R. (2022). Oxygen control: the often overlooked but essential piece to create better *in vitro* systems. Lab. Chip 22, 1068–1092. 10.1039/D1LC00603G 35084420

[B42] Pampaloni F. Reynaud E. G. Stelzer E. H. K. (2007). The third dimension bridges the gap between cell culture and live tissue. Nat. Rev. Mol. Cell Biol. 8, 839–845. 10.1038/nrm2236 17684528

[B43] Park K. M. Gerecht S. (2014). Hypoxia-inducible hydrogels. Nat. Commun. 5, 1–12. 10.1038/ncomms5075 24909742 PMC4161608

[B44] Pattappa G. Johnstone B. Zellner J. Docheva D. Angele P. (2019). The importance of physioxia in mesenchymal stem cell chondrogenesis and the mechanisms controlling its response. Int. J. Mol. Sci. 20, 6. 10.3390/ijms20030484 30678074 PMC6387316

[B45] Patterson A. V. Barham H. M. Chinje E. C. Adams G. E. Harris A. L. Stratford I. J. (1995). Importance of P450 reductase activity in determining sensitivity of breast tumour cells to the bioreductive drug, tirapazamine (SR 4233). Br. J. Cancer 72, (572) 1144–1150. 10.1038/bjc.1995.478 7577460 PMC2033939

[B46] Patterson A. V. Saunders M. P. Chinje E. C. Talbot D. C. Harris A. L. Stratford I. J. (1997). Overexpression of human NADPH:cytochrome c (P450) reductase confers enhanced sensitivity to both tirapazamine (SR 4233) and RSU 1069. Br. J. Cancer 76 (10), 1338–1347. 10.1038/bjc.1997.558 9374381 PMC2228151

[B47] Pepelanova I. Kruppa K. Scheper T. Lavrentieva A. (2018). Gelatin-methacryloyl (GelMA) hydrogels with defined degree of functionalization as a versatile toolkit for 3D cell culture and extrusion bioprinting. Bioengineering 5, 55. 10.3390/bioengineering5030055 30022000 PMC6165498

[B48] Peppas N. A. Hoffman A. S. (2013). “Hydrogels,” in Biomaterials Science (Elsevier), 166–179. 10.1016/B978-0-08-087780-8.00020-6

[B49] Peters K. B. Brown J. M. (2002). Tirapazamine: a hypoxia-activated topoisomerase II poison. Cancer Res. 62, 5248–5253. Available online at: http://www.ncbi.nlm.nih.gov/pubmed/12234992 (Accessed February 16, 2022). 12234992

[B50] Rexius-Hall M. L. Mauleon G. Malik A. B. Rehman J. Eddington D. T. (2014). Microfluidic platform generates oxygen landscapes for localized hypoxic activation. Lab. Chip 14, 4688–4695. 10.1039/C4LC01168F 25315003 PMC4229417

[B51] Rischin D. Peters L. J. O’Sullivan B. Giralt J. Fisher R. Yuen K. (2010). Tirapazamine, cisplatin, and radiation *versus* cisplatin and radiation for advanced squamous cell carcinoma of the head and neck (TROG 02.02, HeadSTART): a phase III trial of the trans-tasman radiation oncology group. J. Clin. Oncol. 28, 2989–2995. 10.1200/JCO.2009.27.4449 20479425

[B52] Sato A. Kadokura K. Uchida H. Tsukada K. (2014). An *in vitro* hepatic zonation model with a continuous oxygen gradient in a microdevice. Biochem. Biophys. Res. Commun. 453, 767–771. 10.1016/j.bbrc.2014.10.017 25445587

[B53] Saunders M. P. Patterson A. V. Chinje E. C. Harris A. L. Stratford I. J. (2000). NADPH:cytochrome c (P450) reductase activates tirapazamine (SR4233) to restore hypoxic and oxic cytotoxicity in an aerobic resistant derivative of the A549 lung cancer cell line. Br. J. Cancer 82 (3), 651–656. 10.1054/bjoc.1999.0977 10682679 PMC2363339

[B54] Schmitz C. Pepelanova I. Seliktar D. Potekhina E. Belousov V. V. Scheper T. (2020). Live reporting for hypoxia: hypoxia sensor–modified mesenchymal stem cells as *in vitro* reporters. Biotechnol. Bioeng. 117, 3265–3276. 10.1002/bit.27503 32667700

[B55] Schmitz C. Pepelanova I. Ude C. Lavrentieva A. (2022). Studies on oxygen availability and the creation of natural and artificial oxygen gradients in gelatin-methacryloyl hydrogel 3D cell culture. J. Tissue Eng. Regen. Med. 16, 977–986. 10.1002/TERM.3344 35962761

[B56] Schuurman W. Levett P. A. Pot M. W. van Weeren P. R. Dhert W. J. A. Hutmacher D. W. (2013). Gelatin-methacrylamide hydrogels as potential biomaterials for fabrication of tissue-engineered cartilage constructs. Macromol. Biosci. 13, 551–561. 10.1002/mabi.201200471 23420700

[B57] Shirahama H. Lee B. H. Tan L. P. Cho N. J. (2016). Precise tuning of facile one-pot gelatin methacryloyl (GelMA) synthesis. Sci. Rep. 6, 1–11. 10.1038/srep31036 27503340 PMC4977492

[B58] Simon M. C. Keith B. (2008). The role of oxygen availability in embryonic development and stem cell function. Nat. Rev. Mol. Cell Biol. 9, 285–296. 10.1038/NRM2354 18285802 PMC2876333

[B59] Spiegelberg L. Houben R. Niemans R. de Ruysscher D. Yaromina A. Theys J. (2019). Hypoxia-activated prodrugs and (lack of) clinical progress: the need for hypoxia-based biomarker patient selection in phase III clinical trials. Clin. Transl. Radiat. Oncol. 15, 62–69. 10.1016/J.CTRO.2019.01.005 30734002 PMC6357685

[B60] Volkmer E. Drosse I. Otto S. Stangelmayer A. Stengele M. Kallukalam B. C. (2008). Hypoxia in static and dynamic 3D culture systems for tissue engineering of bone. Tissue Eng. Part A 14, 1331–1340. 10.1089/ten.tea.2007.0231 18601588

[B61] Von Pawel J. Von Roemeling R. Gatzemeier U. Boyer M. Elisson L. O. Clark P. (2000). Tirapazamine plus Cisplatin *versus* Cisplatin in advanced Non–Small-Cell lung cancer: a report of the International CATAPULT I Study group. J. Clin. Oncol. 18, 1351–1359. 10.1200/JCO.2000.18.6.1351 10715308

[B62] Wagner B. A. Venkataraman S. Buettner G. R. (2011). The rate of oxygen utilization by cells. Free Radic. Biol. Med. 51, 700–712. 10.1016/j.freeradbiomed.2011.05.024 21664270 PMC3147247

[B66] Wenger R. Kurtcuoglu V. Scholz C. Marti H. Hoogewijs D. (2015). Frequently asked questions in hypoxia research. Hypoxia (Auckl) 3, 35–43. 10.2147/HP.S92198 27774480 PMC5045069

[B63] Wan X. Li Z. Ye H. Cui Z. (2016). Three-dimensional perfused tumour spheroid model for anti-cancer drug screening. Biotechnol. Lett. 38, 1389–1395. 10.1007/S10529-016-2035-1 27167883 PMC4942491

[B64] Wolff P. Heimann L. Liebsch G. Meier R. J. Gutbrod M. van Griensven M. (2019). Oxygen-distribution within 3-D collagen I hydrogels for bone tissue engineering. Mater. Sci. Eng. C 95, 422–427. 10.1016/j.msec.2018.02.015 30573266

[B65] Zhu Y. Liu T. Song K. Fan X. Ma X. Cui Z. (2008). Adipose-derived stem cell: a better stem cell than BMSC. Cell Biochem. Funct. 26, 664–675. 10.1002/cbf.1488 18636461

