# Supplementary material for: An oxygen gradient chamber with simple geometry for generating oxygen gradients in hydrogel-based cell cultures within a well-plate-compatible format
Source: Front Bioeng Biotechnol. 2026 Oct 1;14:1937577. doi: 10.3389/fbioe.2026.1937577 (PMC13669165; doi:10.3389/fbioe.2026.1937577)
Supplement: Supplementary file 1 [file DataSheet2.pdf]

Supplementary Material

1 Supplementary Figures

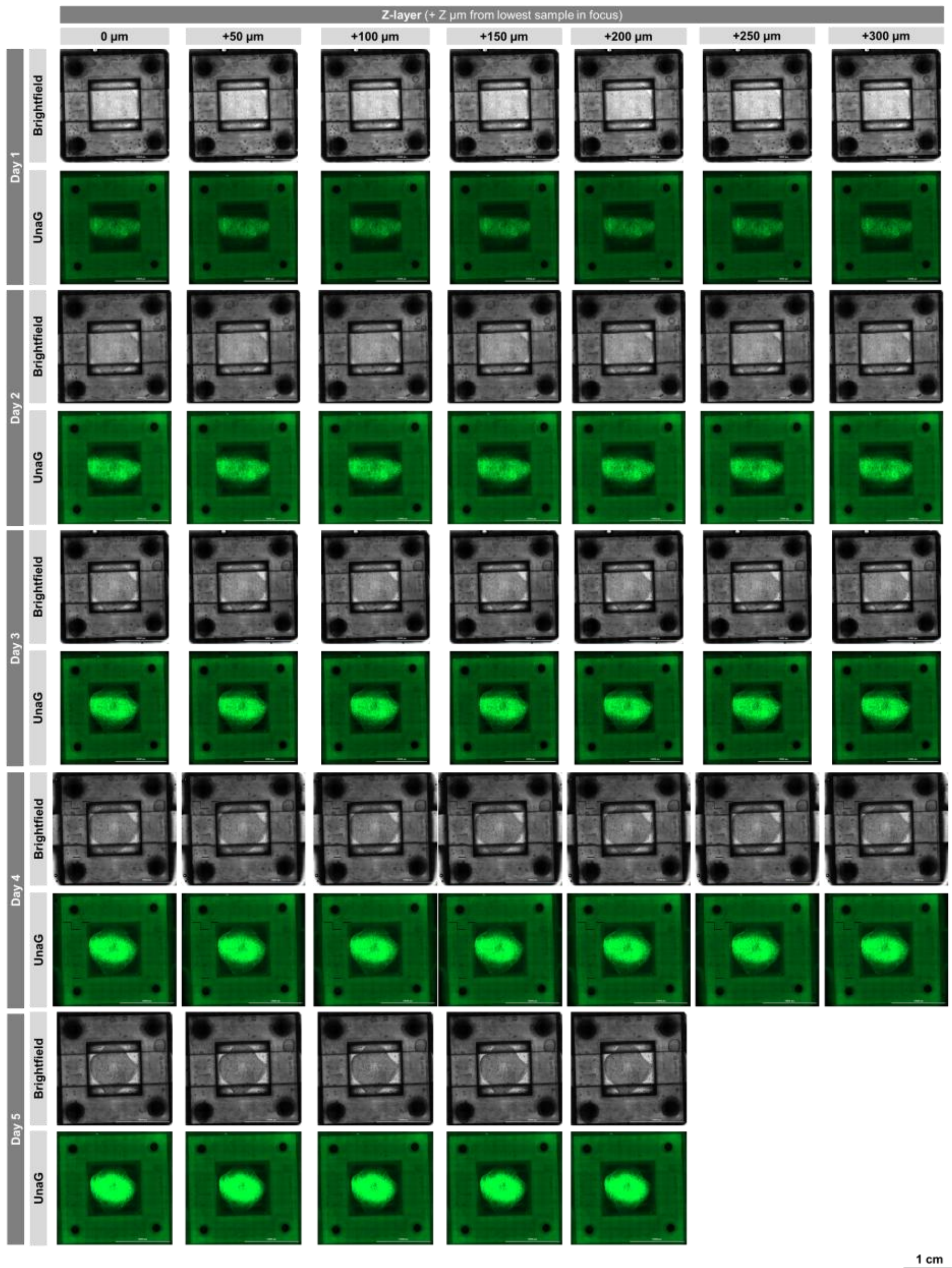

Supplementary Figure 1: Brightfield (top) and fluorescence (bottom) microscopy of hypoxia reporter MSCs in oxygen gradient chamber at day 1-10 after glass mounting. 7.5% w/v low DoF Gelatine methacryloyl seeded with 1×10^6 MSCs per mL of hydrogel. Montage of X-Y axis in different Z-layers (0-300 µm from lowest sample plane in focus) with BioTek Cytation 5. Cells are derived from n=1 donor, and n=1 technical replicate is depicted in this figure. Scale bar: 1 cm.

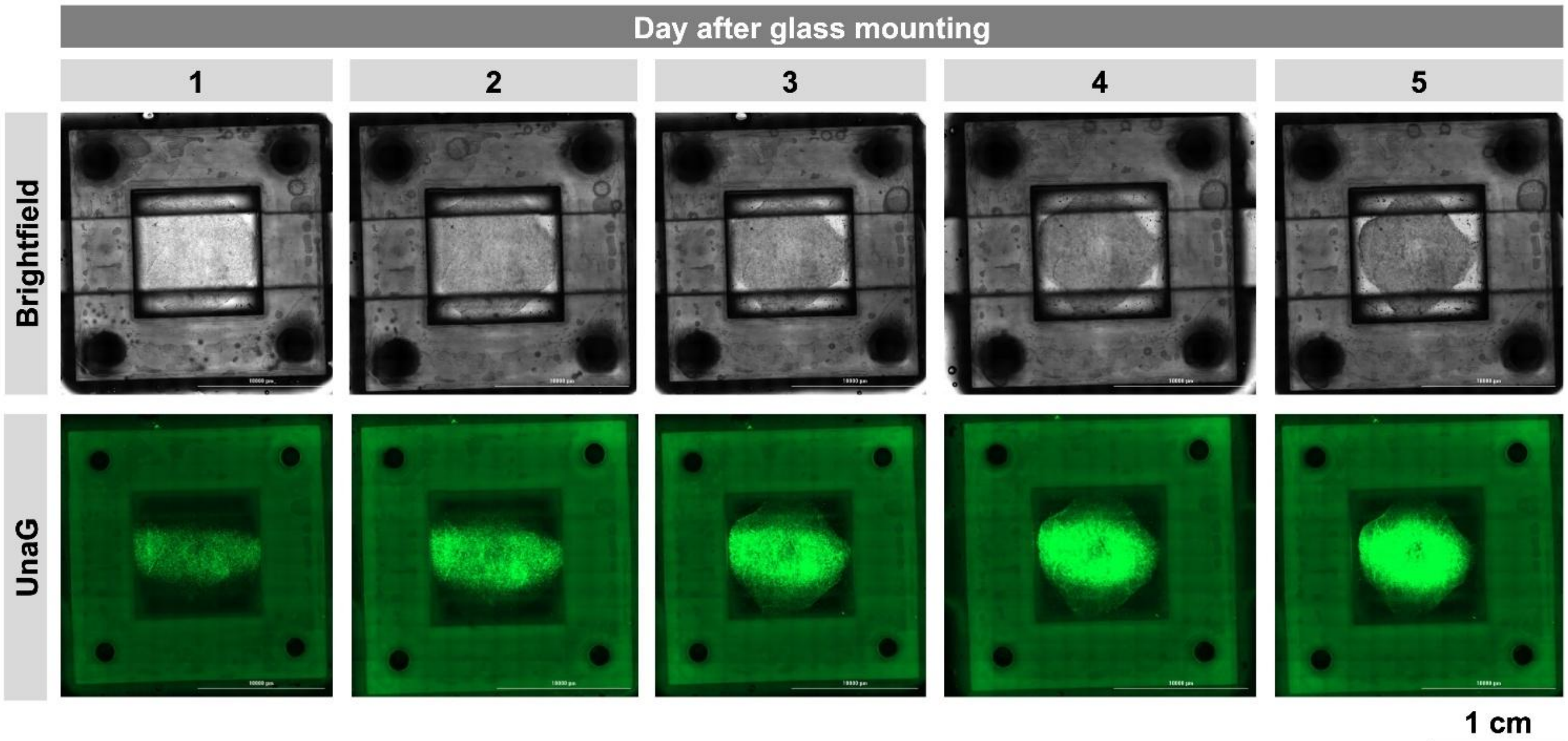

Supplementary Figure 2: Brightfield (top) and fluorescence (bottom) microscopy of hypoxia reporter MSCs in oxygen gradient chamber at day 1-10 after glass mounting. 7.5% w/v low DoF Gelatine methacryloyl hydrogel seeded with 1×10^6 MSCs per mL of hydrogel. Z-stack projection to single image plane acquired with BioTek Cytation 5. Cells are derived from n=1 donor, and n=1 technical replicate is depicted in this figure. Scale bar: 1 cm.

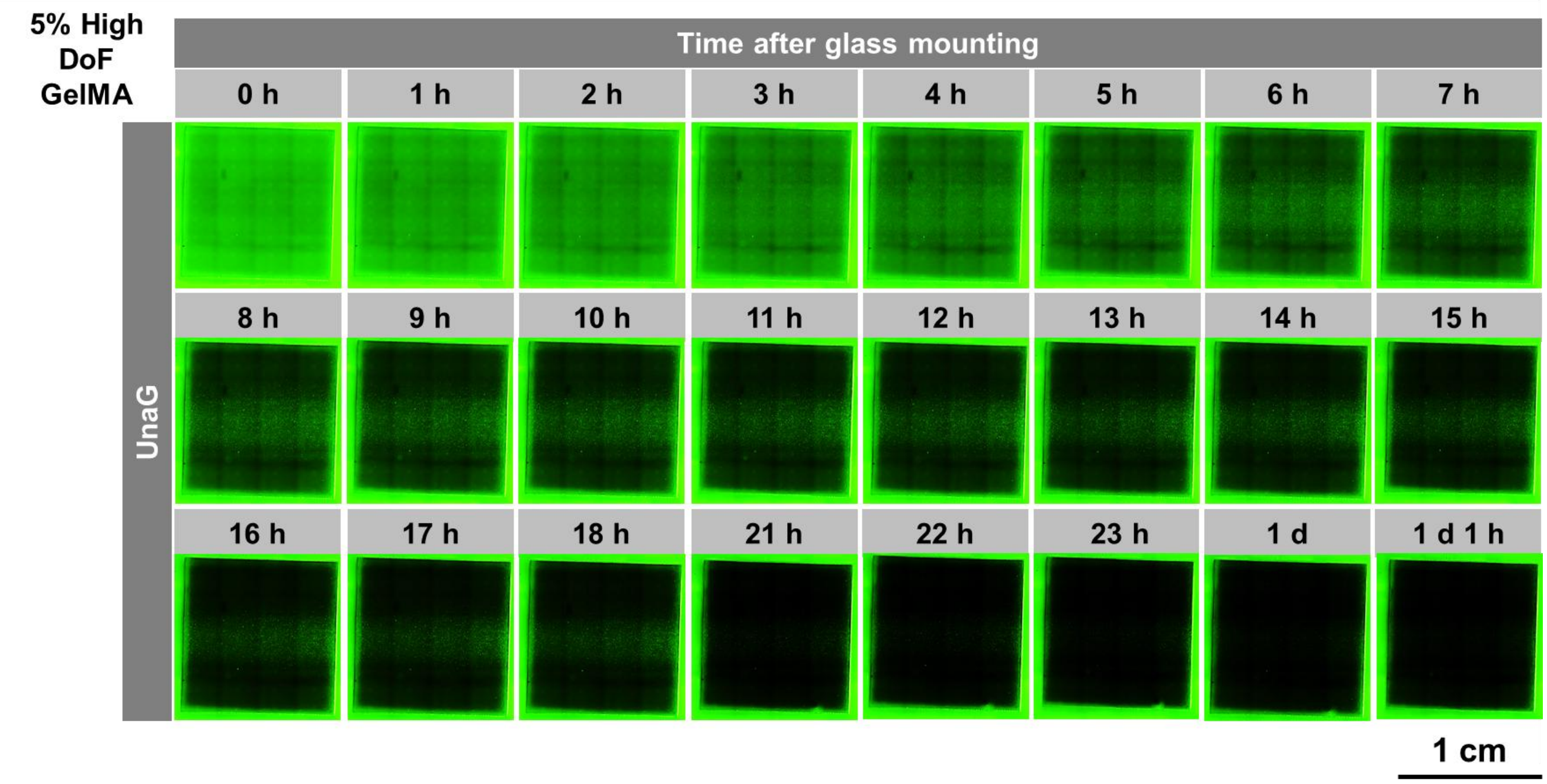

Supplementary Figure 3: Fluorescence microscopy of hypoxia reporter MSCs in oxygen gradient chamber during the first 25 h after glass mounting. 5% w/v high DoF Gelatine methacryloyl hydrogel seeded with 1×10^6 MSCs per mL of hydrogel. Cells are derived from n=1 donor, and n=1 technical replicate is depicted in this figure. Single plane images acquired with Incucyte S3 from a "whole well" scan. Scale bar: 1 cm.

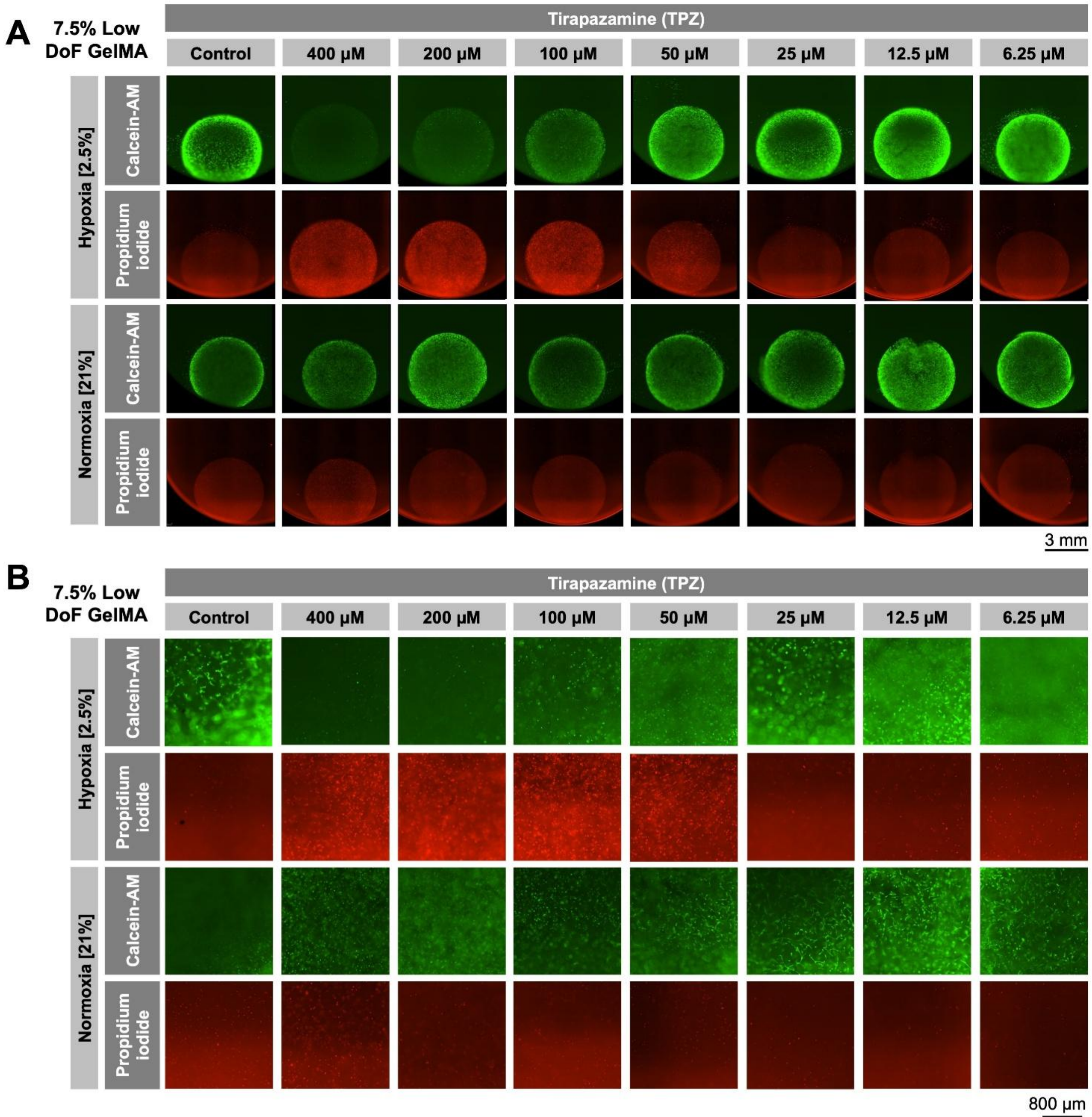

Supplementary Figure 4: Comparison of tirapazamine (TPZ) toxicity at 0-400 µM in 7.5% low DoF 3D hydrogel discs seeded with 1×10^6 MSCs per mL of Gelatine methacryloyl hydrogel after 24 h of hypoxic (2.5% O_2) or normoxic (21% O_2) incubation. Fluorescence microscopy of **A** – full hydrogel construct (Scale bar: 3 mm) and **B** – Representative magnified view of a selected area (Scale bar: 800 µm) after live/dead cell staining with Calcein-AM (green, upper row) and Propidium iodide (red, lower row) after normoxic (21% O_2, upper rows) and hypoxic (2.5% O_2, lower rows) cultivation. One representative image of a total of 3 single-plane images acquired with Incucyte S3. DoF = Degree of functionalisation, GelMA = Gelatine methacryloyl.

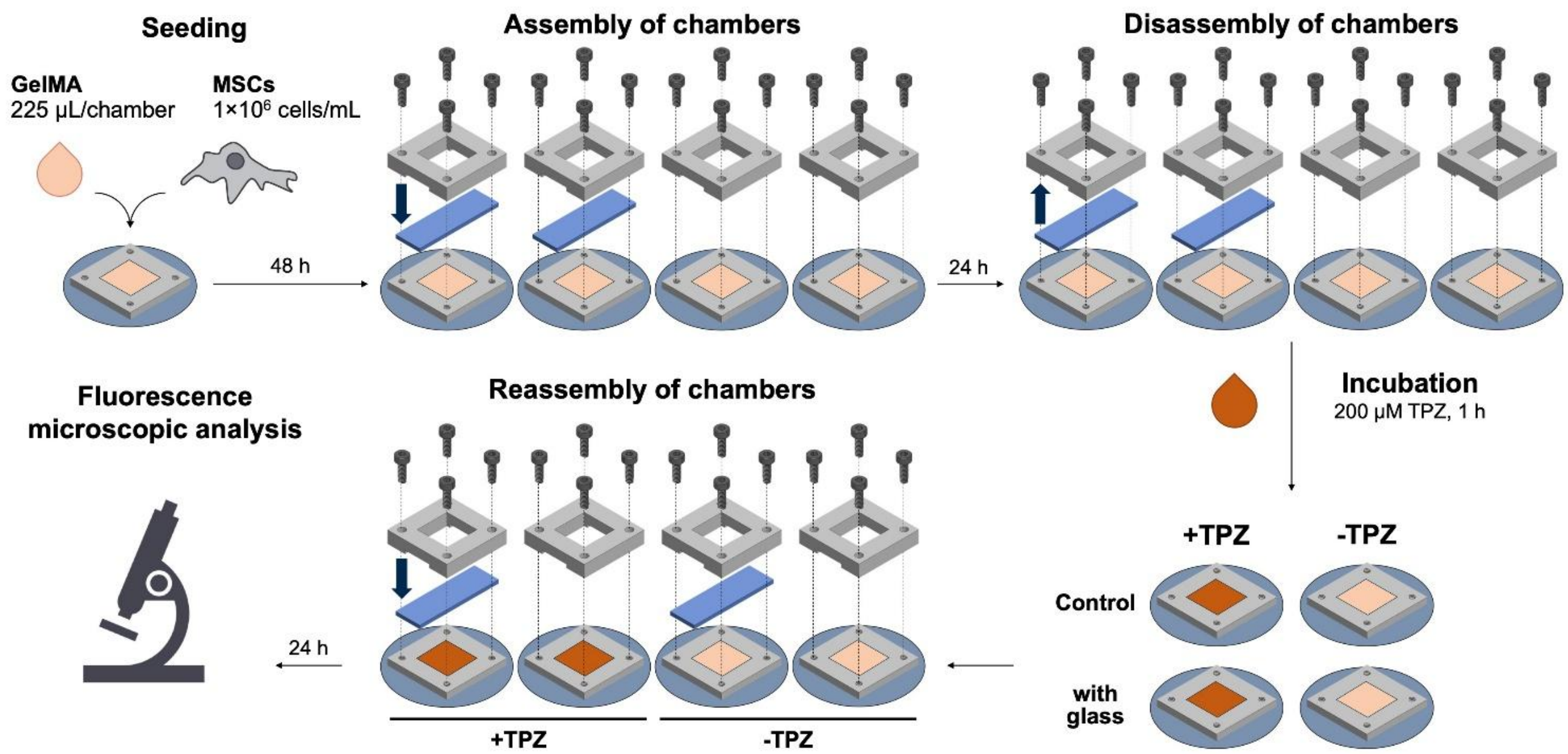

Supplementary Figure 5: Schematic representation of sample treatment during the testing of tirapazamine cytotoxicity at a concentration of 200 µM in the oxygen gradient chamber.

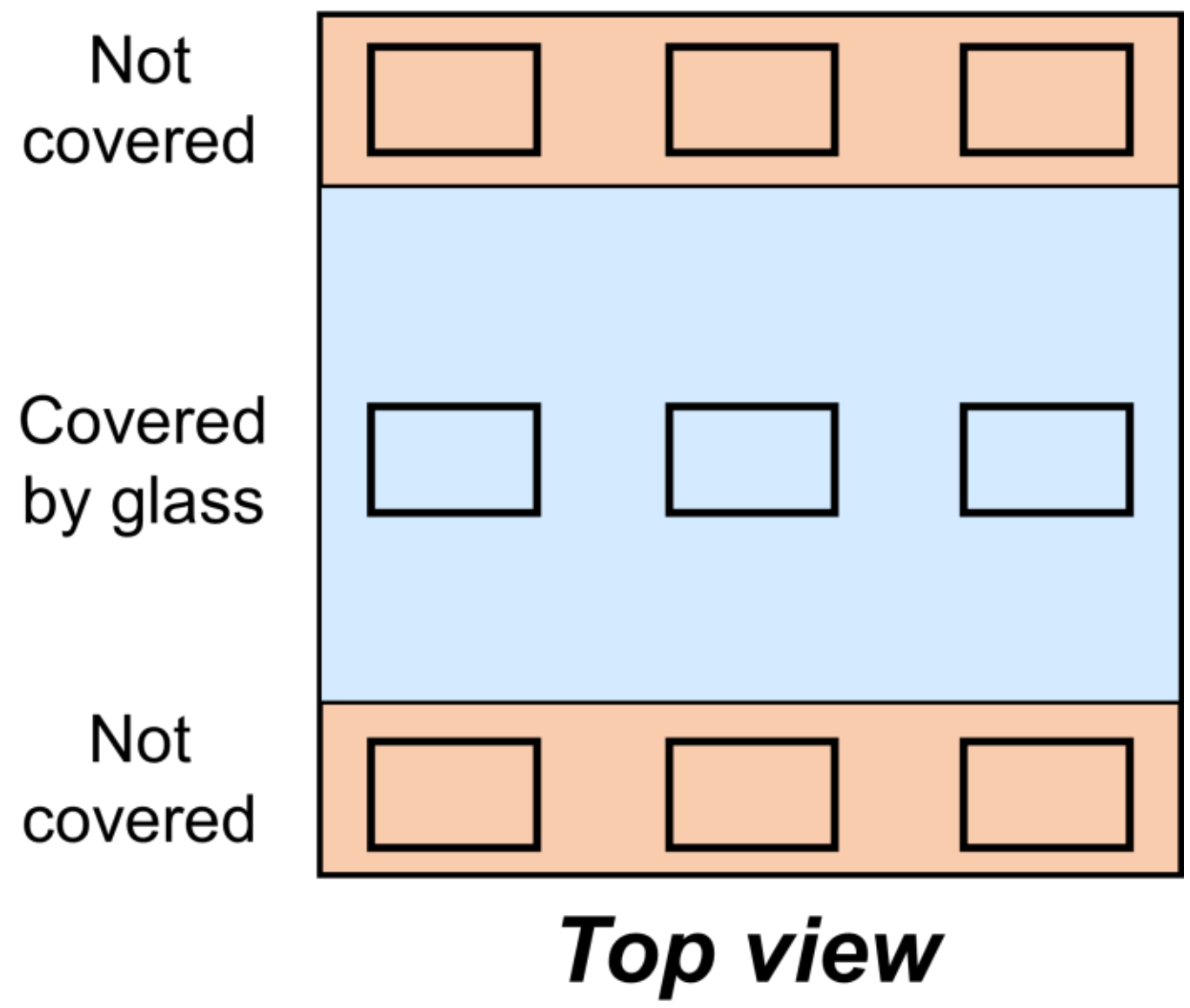

Supplementary Figure 6: Schematic representation of displayed sections of the gradient chamber. Black rectangles depict the area from which a representative image was chosen. Dimension of sections to chamber not up to scale.

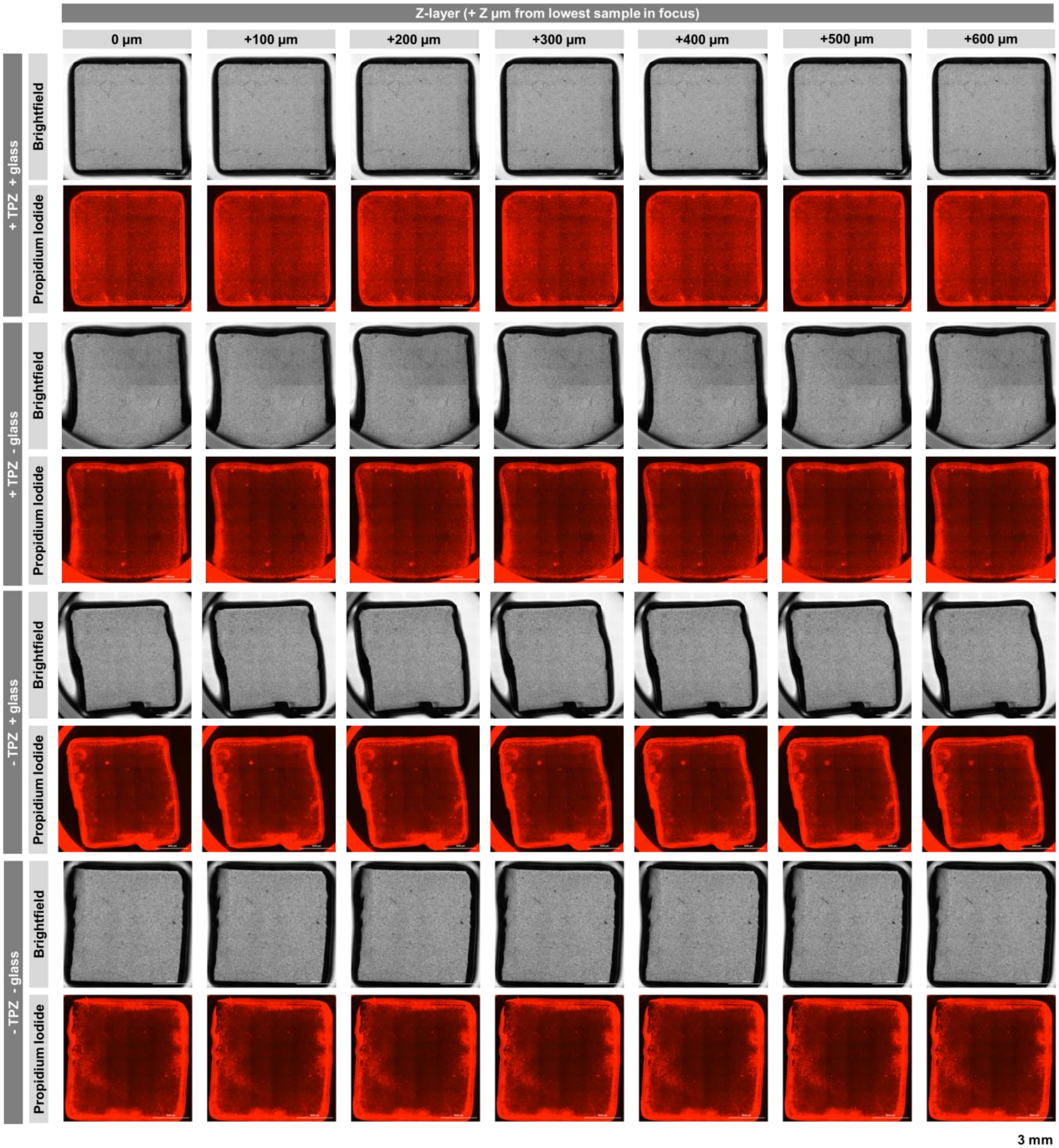

Supplementary Figure 7: Spatially dependent tirapazamine-induced cytotoxicity in the oxygen gradient chamber in different Z-layers (proof-of-concept). Propidium iodide stain of oxygen gradient chambers seeded 1×10^6 MSCs per mL of a 5% high DoF Gelatine methacryloyl hydrogel, cultivated with or without a 6 mm glass cover and treated with or without 200 µM tirapazamine (TPZ) for 24 h. One chamber per condition was analysed as a proof-of-concept experiment. Montage of X-Y axis in different Z-layers (0-600 µm from lowest sample plane in focus) with BioTek Cytation 5. Scale bar: 3 mm.
